# *VE-statin/egfl7* Expression in Endothelial Cells Is Regulated by a Distal Enhancer and a Proximal Promoter under the Direct Control of Erg and GATA-2

**DOI:** 10.1371/journal.pone.0012156

**Published:** 2010-08-16

**Authors:** Alexandra Le Bras, Chantal Samson, Matteo Trentini, Bertrand Caetano, Etienne Lelievre, Virginie Mattot, Friedrich Beermann, Fabrice Soncin

**Affiliations:** 1 CNRS, Institut de Biologie de Lille, UMR 8161, Equipe labellisée La Ligue, Lille, France; 2 Université Lille-Nord de France, Lille, France; 3 Institut Pasteur de Lille, F-59019 Lille, France; 4 Swiss Institute for Experimental Cancer Research (ISREC), Centre de Phénotypage Génomique (CPG), School of Life Sciences, Swiss Federal Institute of Technology (EPFL), Lausanne, Switzerland; Leiden University Medical Center, Netherlands

## Abstract

Angiogenesis is the process by which new blood vessels arise from existing ones by the budding out of endothelial cell capillaries from the luminal side of blood vessels. Blood vessel formation is essential for organ development during embryogenesis and is associated with several physiological and pathological processes, such as wound healing and tumor development. The *VE-statin/egfl7* gene is specifically expressed in endothelial cells during embryonic development and in the adult. We studied here the regulatory mechanisms that control this tissue-specific expression. RT-qPCR analyses showed that the specificity of expression of *VE-statin/egfl7* in endothelial cells is not shared with its closest neighbor genes *notch1* and *agpat2* on the mouse chromosome 2. Chromatin-immunoprecipitation analysis of histone modifications at the *VE-statin/egfl7* locus showed that the chromatin is specifically opened in endothelial cells, but not in fibroblasts at the transcription start sites. A 13 kb genomic fragment of promoter was cloned and analyzed by gene reporter assays which showed that two conserved regions are important for the specific expression of *VE-statin/egfl7* in endothelial cells; a −8409/−7563 enhancer and the −252/+38 region encompassing the exon-1b transcription start site. The latter contains essential GATA and ETS-binding sites, as assessed by linker-scanning analysis and site-directed mutagenesis. An analysis of expression of the ETS and GATA transcription factors showed that Erg, Fli-1 and GATA-2 are the most highly expressed factors in endothelial cells. Erg and GATA-2 directly control the expression of the endogenous *VE-statin/egfl7* while Fli-1 probably exerts an indirect control, as assessed by RNA interference and chromatin immunoprecipitation. This first detailed analysis of the mechanisms that govern the expression of the *VE-statin/egfl7* gene in endothelial cells pinpoints the specific importance of ETS and GATA factors in the specific regulation of genes in this cell lineage.

## Introduction

The endothelium is a monolayer of cells which lines the blood vessel luminal side, in direct contact with the circulation. It forms, in most locations, a tight and selective barrier between the organs and blood. Endothelial cells participate to or regulate thrombosis, leukocyte extravasation, vasodilatation, and angiogenesis. They express most specifically several markers which define their identity. The mechanisms regulating expression of many such markers have been analyzed, such as the VEGF receptor genes *flk-1* [1], [2] and *flt-1* [3], [4], the junction molecule *VE-cadherin* [5], [6] or the receptors *robo-4* [7], [8], *tie-1* [9], [10], and *tie-2* [11], [12]. In most cases, specific gene expression is regulated by a set of discrete binding sites for ETS and GATA factors. Recent data showed that a combination of Forkhead and ETS factors binding sites could also be important for the regulation of endothelial gene promoters [13], [14]. In addition to cis-acting regulation, epigenetic modifications directly participate to the endothelial-specific regulation of gene expression; *eNOS* is highly methylated in non-endothelial cells and is enriched in acetylated H3 and H4 histones in the regions surrounding the promoter and the transcription start site in endothelial cells [15], [16]. *Notch4* is specifically enriched in acetylated histones along its regulatory regions in the first intron and third exon in endothelial cells [17]. Other endothelial genes such as endothelial protein C receptor *epcr* [18] and von Willebrand factor *vwf* [19] are also epigenetically regulated in endothelial cells.

We have initially characterized the *Vascular Endothelial-statin/epidermal growth factor-like 7* (*VE-statin/egfl7)* gene as being specifically and strongly expressed in endothelial cells *in vitro* and *in vivo* [20]. *VE-statin/egfl7* is expressed very early during mouse development; at E7.5, *VE-statin/egfl7* expression is detected exclusively in the primitive blood islands where the first endothelial cells differentiate. Then after during embryogenesis and in the newborn, *VE-statin/egfl7* is expressed in endothelial cells of arteries, veins and capillaries [20], [21]. Expression of *VE-statin/egfl7* decreases in adult tissues, though maintaining its endothelial-specific distribution (Soncin F. not shown, [21]). Of note, *VE-statin/egfl7* expression was also detected at transient and low levels in primordial germ cells [22] and in a subtype of neurons of the mouse adult brain [23] but overall, VE-statin/egfl7 is an excellent marker of the endothelial lineage for which the gene regulation has not been studied in details so far.

*VE-statin/egfl7* encodes a 30kDa secreted protein which represses the migration of smooth-muscle cells in response to PDGF-BB *in vitro* [20]. VE-statin/egfl7 is also an endogenous negative regulator of elastogenesis which prevents the deposition of elastin by endothelial cells [24]. Unfortunately, the reported gene inactivation approaches have been more troublesome than anticipated [25], [26] and we are still in need of a detailed description of the phenotype.

In this study, we describe the first analysis of the regulatory mechanisms that are responsible for the specific expression of *VE-statin/egfl7* in endothelial cells. We identified several important regulatory regions and sites and showed that expression of *VE-statin/egfl7* is directly regulated by the Erg and GATA-2 transcription factors and indirectly by Fli-1.

## Materials and Methods

### Bioinformatics

Comparative genomic analyses and transcription factor binding site prediction were performed using the 30-Way Multiz Alignment & Conservation module of the University of California, Santa Cruz Genome Bioinformatics site (genome.ucsc.edu) and the Genomatix suite (http://www.genomatix.de), respectively. Relevant data were analyzed for statistical significance in the Student's t-test using Analyse-it for Microsoft Excel (version 2.03, Analyse-it Software, Ltd.)

### Cells

H5V [27], EOMA (ATCC CRL-2586), 1G11 [28] and MS1 (ATCC CRL-2279) mouse endothelial cells, or 3T3 (ATCC CRL-1658) and L929 (ATCC CCL-1) fibroblasts were cultured in 78.5 cm^2^ dishes (Falcon) in Dulbecco's Modified Eagle's Medium (Invitrogen), 10% calf or fetal calf serum (Hyclone), 10kU/L penicillin, 10 mg/L streptomycin. Non-essential amino acids and Na-pyruvate were added to 1G11 culture medium. Cells were cultured in a humidified 95% air/5% CO_2_ incubator at 37°C and passaged by standard trypsinisation and 1/5 or 1/10 dilution. Primary human umbilical vein endothelial cells (HUVEC, Lonza) were grown in EGM-2 (Lonza) in 25 cm^2^ ventilated flasks in a humidified 95% air/5% CO_2_ incubator at 37°C. They were passaged by trypsinisation, rinsed in trypsin neutralization solution (TNS, Lonza) and plated at 5000 cell/cm^2^ density. They were used between passage 2 and 7.

### Cloning and mutagenesis

Cloning techniques were essentially as described [29]. A 13.0kb genomic fragment corresponding to the −12969/+38 sequence of the *VE-statin/egfl7* promoter was recovered by NheI/SalI digestion of a mouse genomic clone and inserted into the NheI/XhoI restriction sites of pGL3basic (Promega). Deletion constructs were prepared by ligating the fragments AfeI/SalI (−8409/+38), AflII/SalI (−7563/+38), DraI/SalI (−5133/+38) NdeI/SalI (−2823/+38) and the HpaI/SalI (−1768/+38) into the blunted NheI and the XhoI site of pGL3basic. The −668/+38 region was obtained by digestion of the original mouse genomic fragment with XhoI and SalI and cloned into the XhoI site of pGL3basic in order to generate −668/+38Luc. The −476/+38 region was generated by PCR amplification of the original mouse genomic fragment with the primers 5′-atatgctagccctggttgcacgcagctccag and 5′-aatgtctgctgcccagggtc. The −252/+38 region was generated by PCR amplification of the original mouse genomic fragment with 5′-ata tgc tag cgt ctc tga ctg ctt cag g and 5′-aat gtc tgc tgc cca ggg tc. The −124/+38 region was obtained by digestion of the original mouse genomic fragment with BamHI and SalI. These −476/+38, −252/+38 and −124/+38 fragments were cloned into the NheI and XhoI sites of pGL3basic in order to generate the −476/+38Luc, −252/+38Luc and −124/+38Luc vectors, respectively.

Cloning of −8409/−7563-SV40Luc and −7563/−8409-SV40Luc were performed by digesting the original mouse genomic fragment by AflII/Eco47III and inserting the 846 bp fragment in either orientation in the SmaI site of the pGL3promoter vector (Promega). Cloning of the 7770/−7563-SV40Luc vector was performed by PCR amplification of −8409/−7563-SV40Luc using the 5′-atctcgaggtcatggtgcacaaggaggaag and 5′-atgagctcgtgcctgaccctgaagcctg primers, digestion of the PCR fragments with SacI and XhoI and cloning into the corresponding sites of pGL3promoter.

Site-directed mutagenesis was performed using the overlap extension method [30]. Two separate amplification reactions were first performed using −252/+38Luc (30.1 fmoles) as template; one using the RVprimer3 (Promega, 0.5 µM) and the mutated reverse primer (0.5 µM, Table S1), the other using the mutated forward primer and the GLprimer2 (Promega) with either native Pfu polymerase (Stratagene) or High Fidelity PCR master Mix (Roche) in the recommended buffer in the presence of 5% DMSO when needed. An overlapping reaction was performed using products from the first PCR (2–5%) and the forward and reverse primers. In both instances, thermal conditions were 95°C/5min, 25 cycles [94°C/30s, 55°C/30s, 72°C/30s-1min], 72°C/7min. PCR products were inserted into the NheI/HindIII site of pGL3basic. The −8409/+38EBS6mtLuc and −8409/+38EBS7mtLuc were constructed by exchanging the non-mutated DrdI/SalI fragment of −8409/+38Luc for the corresponding mutated versions of the EBS6 or EBS7 site, respectively. The −8409/+38GATAmtLuc, −8409/+38EBS1mtLuc to −8409/+38EBS5mtLuc vectors were constructed by exchanging a non-mutated BamHI fragment spanning the −8409/+38 sequence and part of pGL3basic from −8409/+38Luc for the corresponding mutated versions of the GATA or of EBS1 to EBS5 sites, respectively. The −8409/+38mtDLuc was constructed by removing the −252 /−54 region from −8409/+38Luc.

### Linker-scanning analysis

***Region A.*** Two separate amplification reactions were performed using −12969/+38Luc (8.6 fmoles) as template; one using a forward primer (0.25µM) and ScanA_n_rev primers (0.25µM, Table S1), the other using the ScanA_n_forward primer and a reverse primer using native Pfu polymerase (Stratagene) in the supplied buffer containing 0.15mM dNTPs and 5% DMSO. Thermal conditions were 95°C/5min, 25 cycles [94°C/30s, 55°C/30s, 72°C/2min], 72°C/7 min. An overlapping PCR was then performed by mixing products from the corresponding first reactions (2%) and the forward and reverse primers. Thermal conditions were 95°C/5min, 5 cycles [94°C/30s, 62°C/30s, 72°C/1.5min], 25 cycles [94°C/30s, 62°C/30s, 72°C/1.5min], 72°C/7 min. PCR products were digested and inserted into the NheI/AflII sites of −12969/+38Luc.

***Region D.*** A similar scheme was applied except that the first amplification reactions were performed using −252/+38Luc (30.1fmoles) as template and the ScanD_n_fwd/GLprimer3 and RVprimer2/ScanD_n_rev primers, respectively (Table S1) using High Fidelity PCR Master Mix (Roche) in the supplied buffer and 5% dimethylsulfoxide. Thermal conditions were 95°C/5min, 33 cycles [94°C/30s, 55°C/30s, 72°C/30s], 72°C/7 min. Overlapping amplifications were performed using the RVprimer3 and GLprimer2 primers. Thermal conditions were 95°C/5min, 33 cycles [94°C/30s, 55°C/30s, 72°C/50s], 72°C/7 min. PCR products were digested and inserted into the NheI/HindIII site of pGL3basic. Vectors were transformed and then produced in Novablue (Novagen) or TG1 bacteria and purified in endotoxin-low conditions (PureLink™ HiPure Plasmid, Invitrogen). Inserts were fully sequenced on both strands (Genoscreen, Lille).

### Transient transfection and transactivation assays

Cells were plated at 15000 cells/cm^2^ in 10cm^2^ plates, grown overnight and transfected with 80 fmoles of the luciferase reporter vector indicated in the figures, 70 fmoles of transactivation vector where indicated, and 54 fmoles pCH110 β-galactosidase normalization vector in OptiMEM (Invitrogen) and in the presence of Exgen 500 reagent (3µl/µg DNA, Euromedex) for 6hr at 37°C in a 5%CO_2_/95% air atmosphere. Total DNA content was adjusted to 1.5µg/well with pUC19. Transfection medium was changed for culture medium and the cells cultured for further 48hr after which they were rinsed with PBS and lysed in Reporter Lysis buffer (Promega). HUVEC were transfected in similar conditions but using Superfect (50µl/µg of DNA, Lonza) as a transfecting agent instead of Exgen-500 and incubating the cells in the presence of DNA for 3h instead of 6h. Cell extracts were assayed for luciferase and β-galactosidase activity using the Luciferase assay system (Promega) and the TROPIX galacto-light β-galactosidase reporter gene assay (Applied Biosystems), respectively. The luciferase value of each sample was normalized with its β-galactosidase value.

### RNA interference

Cells were seeded at 15000cells/cm^2^ in 10cm^2^ dishes, cultured overnight and transfected with 200 to 400pmoles of small interfering RNA (siRNA) designed for each target (Silencer® Select Pre-designed siRNA, Applied Biosystems or ON-TARGETplus SMARTpools, Dharmacon) mixed in 0.5 ml OptiMEM with Lipofectamine 2000 (10µl, Invitrogen) and cultured 48h before RNA extraction and qPCR analysis; Alternatively, cells were lysed in 62.5 mM TRIS-HCl, pH 6.8, 2% SDS, 5% glycerol, 0.28M β-mercaptoEtOH, 0.03% Bromophenol blue, extracts were boiled 3 min before proceeding with SDS-PAGE and Western-blotting.

### Western-blotting

Cell extracts were analyzed by 12% SDS-PAGE and protein transferred onto a PVDF membrane (Immobilon-P, Millipore) overnight at 4°C at 30V under constant stirring of the buffer. Membranes were incubated in PBS, 0.05% Tween-20, 5% non-fat dry milk (Soleil d'Agadir, Lille) for 3h at room temperature and incubated with antibodies directed against VE-statin/egfl7 (1/500, sc-66874), Erg (1/5000, sc-354x, Santa-Cruz), Fli-1 (1/1000, sc-356x, Santa-Cruz), Ets-1 (sc-111x, Santa Cruz), GATA-2 (1/2500, sc-9008x, Santa-Cruz), GATA-4 (1/2500, sc-1237x, Santa-Cruz), or beta-actin (1/1000, sc-1615) in PBS, 0.05% Tween-20, 5% non-fat-dry milk overnight at 4°C under constant mixing, rinsed three times 10 min with PBS, 0.25% Tween-20 at room temperature and further incubated with an horseradish peroxydase-coupled anti-goat, mouse, or rabbit antibody (1/10000, A9452-1VL Sigma, NA931VS, or NA934VS GE-Healthcare, respectively) in PBS, 0.05% Tween-20, 5% non-fat dry milk, rinsed three times in PBS, 0.25% Tween-20 and immunocomplexes were revealed using the Western Lightning-ECL kit (Perkin-Elmer) after exposure to Hyperfilm ECL (GE-Healthcare).

### RNA extraction and reverse transcription

Total RNA were extracted from cells using either Trizol reagent (Invitrogen) or RNeasy kit (Qiagen) and treated with DNaseI reagent (Macherey-Nagel) for 30 min at room temperature. Reverse transcriptions were performed using 1µg total RNA and the High capacity cDNA reverse transcription kit with RNase Inhibitor (Applied Biosystems).

### Chromatin immuno-precipitation

Chromatin immunoprecipitation was performed using Magna-ChIP (Millipore) according to the manufacturer's instructions and the following antibodies: Erg; sc-354x (Santa-Cruz), Fli-1; sc-356x (Santa-Cruz), Ets-1; sc-111x (Santa Cruz), GATA-2 sc-9008x (Santa-Cruz), GATA-4; sc-1237x (Santa-Cruz), acetyl-histone H3; 06-599 (Millipore), dimethyl-histone H3 (Lys4); 07-030 (Millipore), acetyl-histone H4; 06-866 (Millipore), irrelevant antibody; anti-HA.11, MMS-101R (Convance). Briefly, H5V endothelial cells were plated at 15000 cells/cm^2^ and cultured for two days and incubated in culture medium containing 1% formaldehyde for 10 min at 37°C. The reaction was then quenched with 0.125M glycine, the cells were lysed in Lysis buffer containing Protease Inhibitor Cocktail II and disrupted at 4°C using a sonicator (Bioruptor, Diagenode, 20 pulses of 1 min with 1 min intervals) in order to shear the genomic DNA in 1000 bp average-size fragments (not shown). The cell extracts were cleared by centrifugation at 10000g at 4°C for 10 minutes and aliquoted in 50 µl (1.10^6^ cells). For each condition, a 50 µl aliquot was mixed with 450 µl Dilution buffer containing protease inhibitor cocktail II. A 5 µl sample corresponding to INPUT was collected at this point. Extracts were mixed with 5 µg of either antibody and with 20 µl of Magnetic Protein A beads slurry and incubated overnight at 4°C on a rotating wheel. Beads were then washed successively with 0.5 ml Low Salt Immune Complex Wash buffer, High Salt Immune Complex Wash Buffer, LiCl Immune Complex Wash Buffer, and TE Buffer at 4°C. Immunocomplexes were eluted in 100 µl ChIP Elution Buffer containing Proteinase K and incubated for 2 hr at 62°C prior to inactivation at 95°C for 10 min. DNA purification was performed using the provided spin filters, recovered in a 50 µl final volume of Elution Buffer C and stored at −20°C until PCR analysis.

### Quantitative PCR (qPCR)

***qPCR following chromatin immunoprecipitation.*** qPCR were performed in PCR SYBR Green I mix (Roche) containing 10 pmoles of each indicated primer (Table S2). PCR consisted in 40 cycles of [95°C/10s, annealing temperature/10s, 72°C/25s] using the primers and conditions listed in Table S2. Data are expressed as 2^−ΔCt^ where ΔCt = Ct of sample – Ct of INPUT.

***qPCR for VE-statin/egfl7 expression.*** VE-statin/egfl7 and glyceraldehyde 3-phosphate dehydrogenase (GAPDH) transcripts were quantified in duplex FRET PCR reactions using a LightCycler 2.0 (Roche) and the following oligonucleotides: mVE-statin/egfl7 primers 5′-agcatggtttctagtgttg and 5′-ctacggcgataggcagt and fluorescent probes 5′-ggagacctttgtgcagcgt-Fluo and Red640-ataccagccttacctcaccac-3′, GAPDH primers 5′-cttccgtgttcctaccc and 5′-gggtttcttactccttgg and the fluorescent probes 5′-tcctgcgacttcaacagcaac-Fluo and Red705-gccactcttccaccttcgatg-3′. PCR reactions were 95°C/10min, 40 cycles [95°C/10s, 55°C/10s, 72°C/25s]. Alternatively, VE-statin/egfl7 and GAPDH transcripts were quantified in duplex reactions performed in TaqMan Gene Expression Master Mix and the following primers and probe mixes: VE-statin/egfl7; Mm00618004_m1 EGF-like-domain 7 Mus musculus TaqMan assay, beta-actin; Mouse ACTB (actin, beta), GAPDH; mouse GAPDH Endogenous Control (Applied Biosystems); PCR reactions were 50°C/2min, 95°C/10min, 40 cycles [95°C/15s, 60°C/1 min]. Levels were calculated and compared using the ΔΔCt method.

***qPCR of GATA and ETS factors, notch1 and agpat-2*** **.** Oligonucleotides were designed using the Primer Express II software (Applied Biosystems) in order to amplify regions of the cDNA based on exon-exon junctions and so as to cover at least two exons (Table S3). qPCR of cDNA were carried out using Power SYBR Green PCR Master Mix with the following cycles: 95°C/10 min, 40 cycles [95°C/15s, 60°C/1 min] using a StepOne machine (Applied Biosystems). A melting curve was determined after each reaction. For each gene, conditions were setup in order to ensure linearity of amplification over the ranges analyzed.

## Results

### Analysis of the chromosome 2 region encompassing the *VE-statin/egfl7* locus

**Figure 1:**
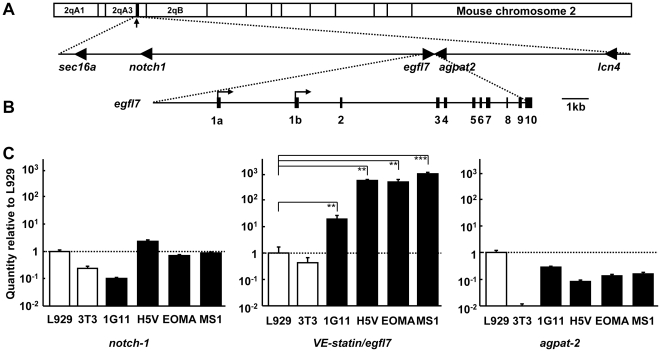
Structure of the *VE-statin/egfl7* gene and promoter. **A**. Top: schematic representation of the distal region of the mouse chromosome 2. The region encompassing the *VE-statin/egfl7* gene is indicated with an arrow. Bottom: distribution of the genes along this region of the chromosome represented to scale, arrowheads indicate the orientation of the genes. **B**. Scaled schematic representation of the *VE-statin/egfl7* gene. Arrows indicate the previously identified transcription start sites [20], exons are represented as numbered black boxes. **C**. Expression levels of *notch1*, *VE-statin/egfl7*, and *agpat2* were measured in cultured L929 and 3T3 fibroblasts (white bars), and in 1G11, H5V, EOMA, and MS1 endothelial cells (black bars) after total RNA isolation and RT-qPCR. Levels of each transcripts were normalized to that of GAPDH measured in the same sample and represented as fold over mean levels in L929 cells set to 1. Y-axis scales are Log10 representations. *** p<0.001, ** p<0.01, * p<0.05.

The *VE-statin/egfl7* gene is located on the mouse chromosome 2 (Figure 1A) and is composed of 11 exons. Exon-1a and -1b are initiated from two different transcription start sites (Figure 1B), the transcript -1b being the most abundant in endothelial cells [20]. In order to assess whether the *VE-statin/egfl7* gene could be located in a larger cluster of endothelial-specific genes on chromosome 2, the expression levels of *VE-statin/egfl7* and that of its flanking neighbors *notch1* and *agpat2* were measured. As already observed [20], *VE-statin/egfl7* expression levels are several tens- to hundreds-fold higher in endothelial cells than in fibroblasts (Figure 1C). *Notch1* is located 116 kbp ahead of the 5′ end of *VE-statin/egfl7* and is expressed in several tissues during mouse development, including, but not specifically, in endothelial cells, it plays an important role in angiogenesis [31], [32]. *Notch1* is not up-regulated in endothelial cells as it is expressed at similar levels in these cells and in fibroblasts (Figure 1C). *Agpat2* is located 2 kbp after the 3′ end of *VE-statin/egfl7*, it is mostly expressed in adipocytes [33], [34]. *Agpat2* is expressed at an average 10-times lower levels in endothelial cells when compared to L929 fibroblasts, its expression levels are almost non-detectable in 3T3 cells (Figure 1C). *VE-statin/egfl7* is thus not located in a cluster of genes highly expressed in endothelial cells, suggesting that it holds its own regulatory signals.

**Figure 2:**
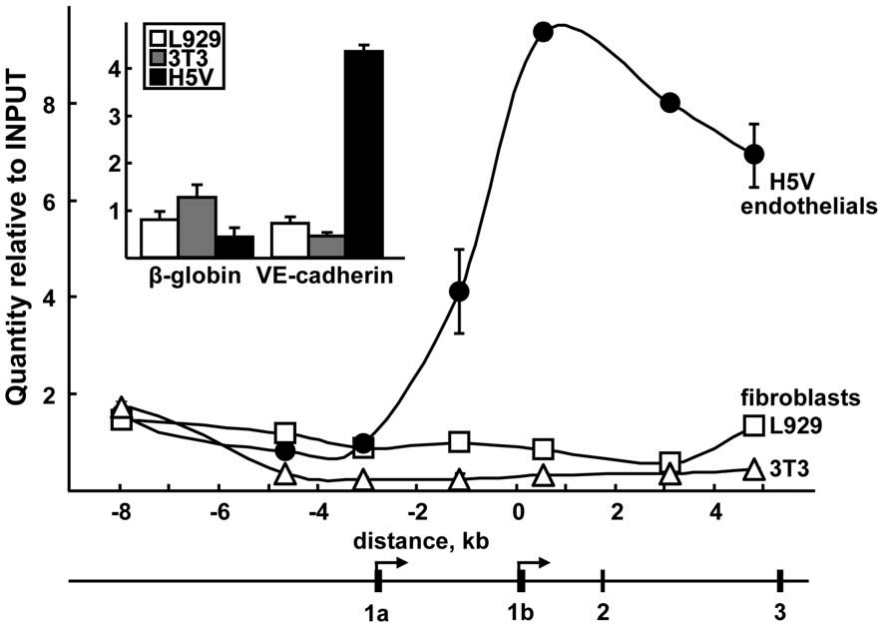
Acetylated Histone H3 levels along the *VE-statin/egfl7* promoter. **Top.** Levels of acetylated-histone H3 along the *VE-statin/egfl7* gene were quantified using chromatin immunoprecipitation of 1% formaldehyde-treated cultures of H5V endothelial (•, black bars), L929 (□, white bars), and 3T3 (Δ, grey bars) fibroblast DNA followed by sonication, immunoprecipitation of the DNA-protein complexes using an acetyl Histone-H3 antibody (06-599, Millipore), DNA purification and semi-quantitative PCR analysis performed in order to amplify various locations along the promoter represented along the x-axis. Quantities are relative to the diluted INPUT mean value set to 1. Acetylation levels were low in all cells in the regions located ahead of the transcription start -1b, they were elevated only in endothelial cells thereafter. **Inset:** Acetylated-histone H3 levels of the negative control *β-globin* and the positive control *VE-cadherin* gene promoters taken as non- and highly-expressed genes in endothelial cells, respectively, and assessed in similar conditions. **Bottom.** Scale schematic representation of the mouse *VE-statin/egfl7* promoter and gene, scaled according to the x-axis in A. Arrows indicate the previously identified transcription start sites [20], exons are represented as numbered black boxes.

Analysis of the global chromatin structure in the 5′ region of the *VE-statin/egfl7* gene showed that the levels of acetylated-histone H3 (Figure 2A), acetyl-histone H4 and dimethyl-histone H3 (Lys 4) (Figure S1) were low in all cells in the regions comprised between −8.0kb and −3.0kb ahead of the transcription start site -1b. The levels then increased in endothelial cells starting at −1kb of the exon-1b and thereafter, while they remained low in fibroblasts. Altogether combined, these analyses of histone modifications show that the chromatin is opened at the *VE-statin/egfl7* locus in endothelial cells and condensed in non-endothelial cells. Accordingly, a treatment of with the histone deacetylase inhibitor Na butyrate induced a strong rise of *VE-statin/egfl7* expression levels in non-endothelial cells (Figure S2).

### Deletion-analysis study of the 5′ region of the gene

Highly conserved promoter regions are more likely to contain important sequences for gene regulation [35]. An alignment and conservation analysis of human and mouse sequences focusing on the *VE-statin/egfl7* gene regions located ahead of the transcription starts -1a and -1b showed the presence of several well-conserved regions, noted A to E (Figure S3). These predicted conserved regions are located at −7755/−7644 (region A), −5414/−5174 (B), and −2829/−2659 (C, which encompasses exon-1a), −253/+68 (D) and +3009/+3068 (E) from the exon-1b transcription start site.

**Figure 3:**
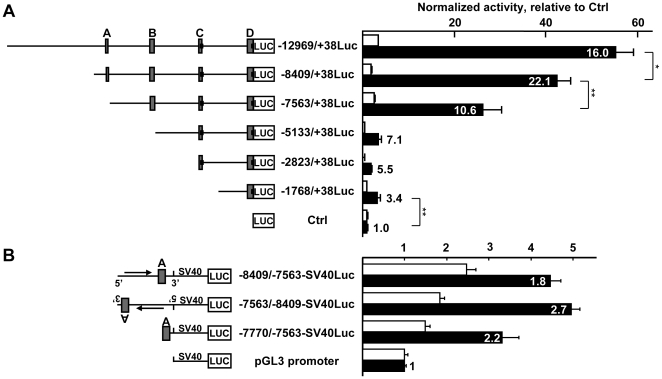
5′-deletion study of the *VE-statin/egfl7* promoter region. **A**. H5V endothelial (black bars) and L929 fibroblast (right, white bars) were transfected with the pGL3basic luciferase reporter vector (Ctrl) or pGL3basic in which −12969/+38 *VE-statin/egfl7* promoter region or 5′ deletions of it were inserted. These reporters (80 fmoles) were transfected together with 54 fmoles pCH110 normalization vector. After 48h of culture, cells were lyzed and the luciferase value of each sample was measured and normalized with its β-galactosidase value. Bars represent normalized activity as fold over Ctrl mean value set to 1. Numbers associated with the black bars represent the calculated endothelial/fibroblast ratio of activity for the corresponding construct. Left: scaled schematic representation of the constructs, names are given according to the cloned 5′ and 3′ end positions relative to the exon-1b transcription start, Luc; luciferase. The experiment is representative of a set of at least three experiments performed in similar conditions. Constructions were designed so that the luciferase gene was placed within exon-1b because initial experiments showed that placing it where the coding sequence starts in exon-3 resulted in no detectable activity (not shown). ** p<0.01, * p<0.05. **B**. H5V endothelial cells (black bars) or 3T3 fibroblasts (white bars) were transfected with 80 fmoles of pGL3promoter (Ctrl, Promega), −8409/−7563SV40Luc, −7563/−8409-SV40Luc, or −7770/−7563-SV40Luc and with 54 fmoles of pCH110 normalization vector. After 48h of culture, cells were lyzed and the luciferase value of each sample was measured and normalized with its β-galactosidase value. Bars represent normalized activity as fold over Ctrl mean value set to 1. Results are displayed as in Figure 3A. The −8409/−7563 fragment is active in endothelial cells regardless of orientation, the most active sequence in this fragment corresponds to region A (−7770/−7563) which shows an endothelial/fibroblast ratio similar to the reporter containing the whole −8409/−7688 region.

A 13kb genomic fragment corresponding to the sequence located ahead of exon-1b was isolated and used to construct a series of luciferase reporter vectors following a 5′-deletion strategy based on the location of the predicted conserved regions. These vectors were then assessed for basal activity in endothelial cells and in fibroblasts by transient transfection of H5V and L929 cells, respectively. The strongest activity levels in both cell types were obtained when the conserved regions A to D were present in the reporters, such as in −12969/+38Luc and −8409/+38Luc, but with a much higher activity in endothelial cells than in fibroblasts, leading to a high endothelial/fibroblast ratio of activity (Figure 3A). In endothelial cells, −8409/+38Luc was the most active construct, displaying almost as much activity as the longest −12969/+38Luc but with a higher endothelial/fibroblast ratio (22.1). Removing the −8409/−7563 fragment, which includes region A, induced a 38% loss of response in endothelial cells (−7563/+38Luc) and a drop of the endothelial/fibroblast ratio by half (10.6) when compared to −8409/+38Luc. Further deletion of 2.4 kb including region B (−5133/+38Luc) induced another marked drop of activity in both endothelial cells and fibroblasts, which lost 92% and 75% of that obtained with −8409/+38Luc, respectively. However, the endothelial/fibroblast ratio remained high (7.1), suggesting that all the important features for expression of the gene in endothelial cells had not been lost. Further removal of 2.3 kb (−2823/+71Luc) induced another decrease of the remaining activity by half with no major incidence on the remaining endothelial/fibroblast ratio. The shortest construct (−1768/+38Luc) displayed a low but yet detectable activity in endothelial cells, corresponding to a 93% loss of that in −8409/+38Luc but still with an endothelial/fibroblast ratio of 3.4.

Similar results were obtained when these promoter constructs were tested in human primary HUVEC endothelial cells (Figure S4), suggesting that the regulatory signals are conserved between human and mouse endothelial cells.

### The −8409/−7563 region encompassing region A is an autonomous enhancer of transcription

Further analysis of the −8409/−7563 sequence showed that it acts as an enhancer of transcription in endothelial cells, as it is able to activate a minimal promoter reporter regardless of orientation (Figure 3B). Region A (−7770/−7563) is the most active region in this fragment. Despite a linker-scanning analysis of the whole area (Figure S5) and numerous site-directed mutations (not shown), no more precise parts of this region were found to be necessary for a strong activity of the promoter in endothelial cells.

### Analysis of the proximal promoter - importance of region D (−252/+38)

**Figure 4:**
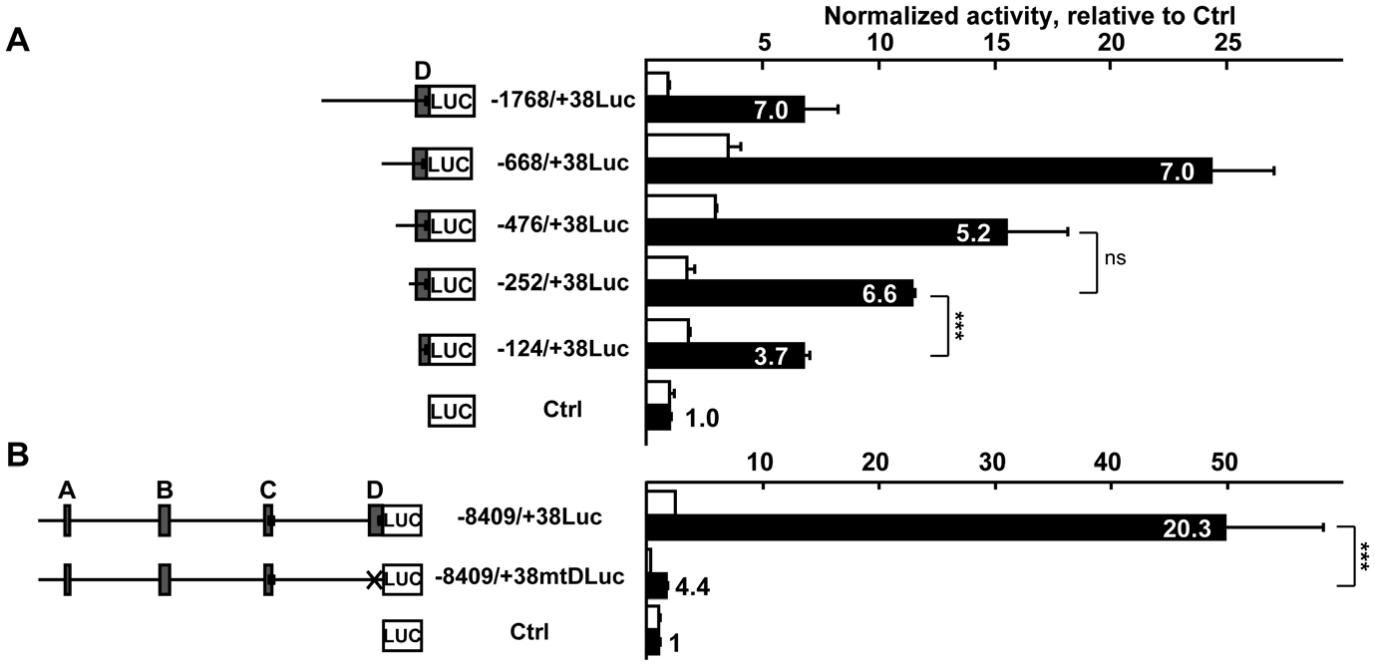
Region D is necessary for expression of the gene in endothelial cells. **A**. Right: H5V endothelial cells (black bars) and L929 fibroblasts (white bars) were transfected with the pGL3basic vector (Ctrl) or successive 5′-deletion mutants of the −1768/+38Luc vector cloned into pGL3basic (80 fmoles) and with 54 fmoles of pCH110 normalization vector. After 48h of culture, cells were lyzed and the luciferase value of each sample was measured and normalized with its β-galactosidase value. Bars represent normalized activity as fold over Ctrl mean value set to 1. Results are presented as in Figure 3A. The experiment is representative of a set of three performed in similar conditions. *** p<0.001, ns; not significant. The most active sequence in the proximal promoter corresponds to the conserved region D within the −1768/+38 fragment. **B**. H5V endothelial cells (black bars) and L929 fibroblasts (white bars) were transfected with 80 fmoles pGL3basic (Ctrl), −8409/+38Luc or the −8409/+38mtDLuc reporter where region D had been removed and with 54 fmoles pCH110 normalization vector. After 48h of culture, cells were lyzed and the luciferase value of each sample was measured and normalized with its β-galactosidase value. Bars represent normalized activity as fold over Ctrl mean value set to 1. Results are represented as in Figure 3A. The experiment is representative of a set of three performed in similar conditions. *** p<0.001. While the −8409/+38 region provided high expression levels in endothelial cells when compared to fibroblasts, most of its activity was lost when region D was removed from this construct.

The subsequent analysis was thus focused on the more proximal −1768/+38 sequence of the gene. A progressive 5′ deletion approach was performed based on the proximal −1768/+38 sequence of the gene which comprises the conserved region D. Removal of 1.1 kb (−668/+38Luc) induced a strong increase of activity both in endothelial and non-endothelial cells but did not affect the endothelial/fibroblast ratio when compared to −1768/+38Luc (Figure 4A), suggesting the presence of a strong inhibitory sequence in the −1768/−668 region. Further removal of 0.19 kb (−476/+38Luc) and of an additional 0.22 kb (−252/+38Luc) did not markedly affect the endothelial/fibroblast ratio (5.2 and 6.6) though it progressively decreased the global activity when compared to −668/+38Luc. On the other hand, the further deletion of 128 bp from the 5′-end of region D (−124/+38Luc) affected both the activity, which decreased by 41%, and the endothelial/fibroblast ratio, which decreased from 7.0 to 3.7 when compared to −252/+38Luc.

Interestingly, removal of the conserved region D from the most active −8409/+38 sequence resulted in an almost completely silent −8409/+38mtDLuc reporter, with activity dropping 97% in endothelial cells while the endothelial/fibroblast ratio fell from 20.3 to 4.4 when compared to −8409/+38Luc (Figure 4B), confirming that region D plays a crucial role for expression of the gene in endothelial cells.

### Identification of the essential sequences within region D

**Figure 5:**
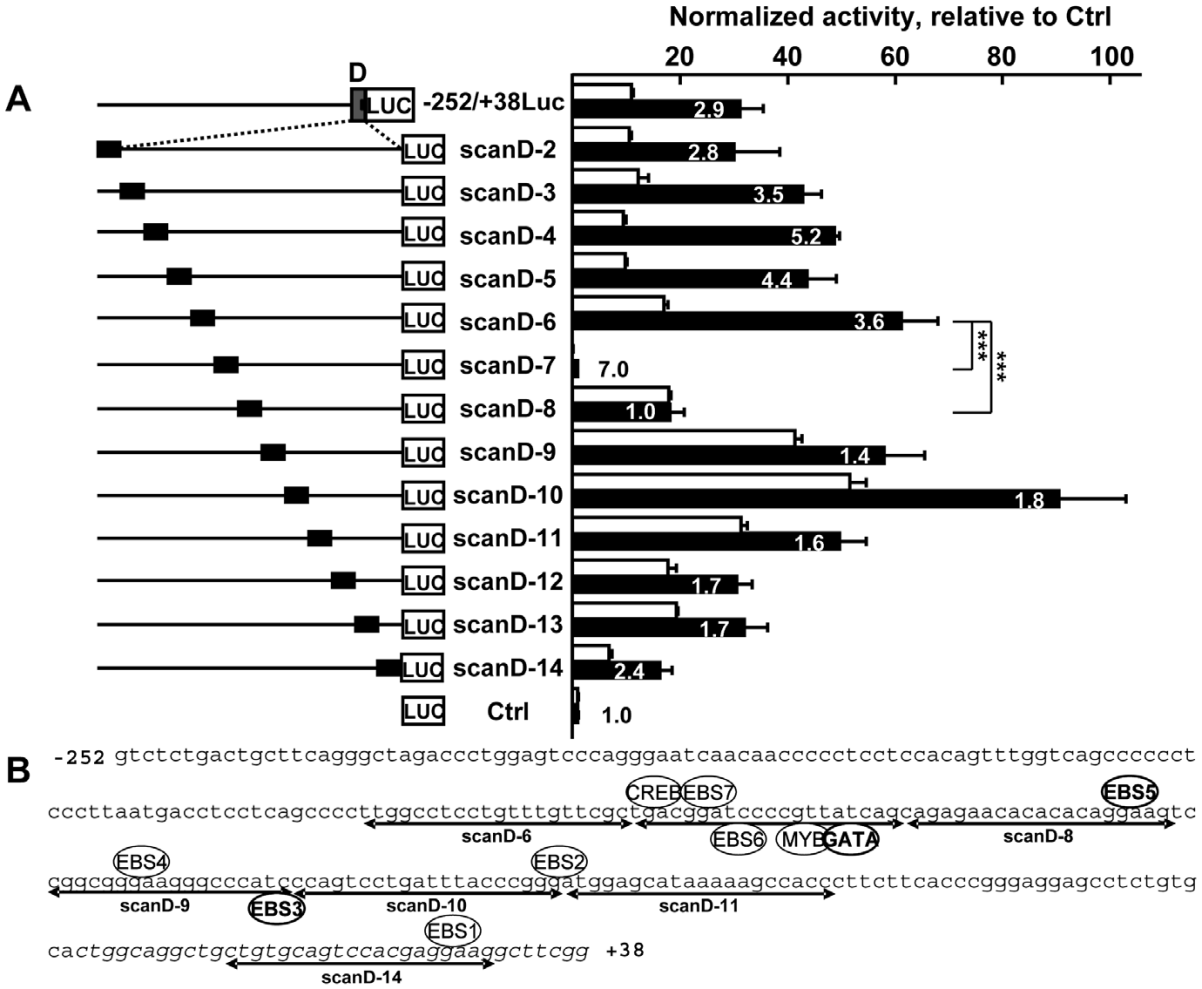
Identification of important sequences in region D. **A**. H5V endothelial cells (black bars) and L929 fibroblasts (white bars) were transfected with 80 fmoles of either pGL3basic (Ctrl), the non-mutated −252/+38Luc construct, or various mutated versions of it (scan D-2 to 14) which corresponded to the successive exchange of 20bp of wild-type sequence for a 20bp transactivation-null cassette (black box), and with 54 fmoles of pCH110 normalization vector. After 48h of culture, cells were lyzed and the luciferase value of each sample was measured and normalized with its β-galactosidase value. Bars represent normalized activity as fold over the Ctrl mean value set to 1. Results are displayed as in Figure 3A. The experiment is representative of a set of three experiments performed in similar conditions. *** p<0.001. Mutation of the sequences corresponding to scanD-7 and scanD-8 greatly affected the activity of the reporters, suggesting the presence of essential sites within these sequences. **B.** Sequence and schematic representation of all the predicted transcription factor binding sites in the scanD-7 and scanD-8 sequence, and of the EBS in the −252/+38 region of the gene. The 5′ part of exon-1b is italicized. The sequences corresponding to the scanD-n mutants are underlined with double arrows. The predicted binding sites which are conserved among species (Figure S6) are in bold.

In order to determine which parts of region D allowed high expression levels in endothelial cells, a linker-scanning analysis of its entire sequence was performed. This analysis corresponded to the transfection of cells with a series of mutants of −252/+38Luc where a stretch of 20 bases was exchanged for a transcriptionnaly-inactive sequence successively along the whole region [36]. Mutation of the sequences located between −129 and −109 (scanD-7) and between −109 and −89 (scanD-8) induced a 99% and a 70% drop in activity in endothelial cells when compared to the scanD-6 levels, respectively (Figure 5A). Further, the endothelial to fibroblast ratio was abolished with the scanD-8 mutant. This suggested that i) the scanD-7 and scanD-8 contain essential sites for activation of the promoter in endothelial cells and in fibroblasts, and ii) the scanD-8 region contains sites that are specifically important for expression of the reporter in endothelial cells.

### Identification of individual functional sites within region D

A computer analysis using the Genomatix suite performed on the −252/+38 sequence predicted the presence of one putative CREB (−127), one MYB-binding site (−116), one GATA site (−113) and an EBS head-to-head tandem (EBS6/EBS7, −123/−120) in the scanD-7 sequence, and one EBS (EBS5, −92) in scanD-8 (Figure 5B). Among these, the CREB, MYB and EBS6/7 tandem sites are not conserved within mammals whereas the GATA and EBS5 are perfectly conserved in all analyzed species, including fishes (Figure S6). Among the sites that are not within scanD-7 or scanD-8, EBS3 is perfectly conserved among species whereas EBS4 is conserved with a functional substitution (GGA*A* instead of GGA*T*) in chicken, stikleback, fugu, and medeka or a one-base shift in marmoset (Figure S6). EBS3 and EBS4 are within the scanD-9 sequence.

**Figure 6:**
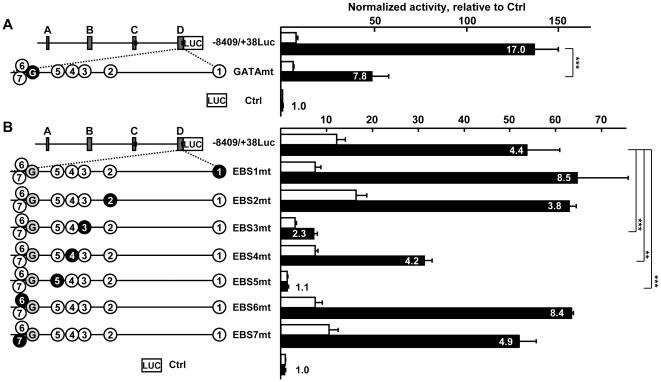
Mutation analysis of GATA and ETS sites in the −252/+38 region of the promoter. **A**. Right; H5V endothelial cells (black bars) and L929 fibroblasts (white bars) were transfected with 80 fmoles of pGL3basic (Ctrl), −8409/+38Luc or a −8409/+38Luc mutant where the GATA (GATAmt) site had been mutated, and with 54 fmoles pCH110 normalization vector. After 48h of culture, cells were lyzed and the luciferase value of each sample was measured and normalized with its β-galactosidase value. Bars represent normalized activity as fold over Ctrl mean value set to 1. Mutation of the GATA site (left, blackened circle) in the originally most active −8409/+38Luc vector resulted in a strong decrease of activity of the reporter in endothelial cells and the endothelial/fibroblasts ratio was decreased by half when compared to −8409/+38Luc. The experiment is representative of a set of three experiments performed in similar conditions. **B**. Right; H5V (black bars) and L929 (white bars) cells where transfected with 80 fmoles of −8409/+38Luc or the indicated mutated versions in which the predicted EBS (left, numbered circles) were individually mutated by site-directed mutagenesis (left, blackened numbered circles). After 48h of culture, cells were lyzed and the luciferase value of each sample was measured and normalized with its β-galactosidase value. Bars represent normalized activity as fold over Ctrl mean value set to 1. Left; scaled schematic representation of the −8409/+38 wild-type region (top). The location of the GATA (circled G) and EBS (circled numbers) sites is represented to scale along the zoomed region D of the mutants. Mutated sites are represented as blackened circles. Names of the constructs are given according to the cloned 5′ and 3′ end positions relative to the exon-1b transcription start, Luc; luciferase. Results are presented according to Figure 3A. The EBS6/EBS7 (−123/−120) tandem is located in scanD-7 of the linker scanning analysis, EBS5 (−92) is located in scanD-8 and EBS4 (−80), EBS3 (−71), EBS2 (−49) and EBS1 (+28) are all located closer to the transcription start. The experiment is representative of a set of three performed in similar conditions. *** p<0.001, ** p<0.01.

Experimental mutation of the GATA site induced a 64% and a 22% loss of activity of the initially most active −8409/+38Luc reporter in endothelial and fibroblast cells, respectively (Figure 6A). The most striking effects were obtained when EBS5 was mutated in the originally most active −8409/+38Luc, resulting in an almost inactive reporter, with a 97% and 89% decrease of activity in endothelial cells and fibroblasts, respectively (Figure 6B). This 2-bases mutation also abolished the endothelial/fibroblast ratio. This mutation alone could thus account for the effects observed with the scanD-8 mutant (Figure 5A) or with the deletion of the entire region D in the full length reporter (Figure 4B). Mutation of EBS3 markedly affected activity, which dropped 87% and 75% in endothelial cells and fibroblasts, respectively, thus decreasing the endothelial/fibroblast ratio when compared to −8409/+38Luc. Mutation of EBS4 reduced the activity of the reporter by 41% and 38% in endothelial cells and fibroblasts, respectively. Mutation of EBS1 or of EBS2 did not significantly affect activity in endothelial cells, although mutation of EBS1 doubled the endothelial/fibroblast ratio when compared to −8409+/38Luc mainly because of a drop of activity in fibroblasts. Mutation of EBS6 or of EBS7 did not significantly affect activity in endothelial cells although mutation of EBS6 doubled the endothelial/fibroblast ratio when compared to −8409/+38Luc, as observed with the EBS1 mutant.

### Erg, Fli-1 and GATA-2 are expressed at high levels in endothelial cells

The previous results pinpointed the potential importance of ETS and GATA transcription factors in the regulation of the *VE-statin/egfl7*. In an attempt to identify the involved transcription factors among these families, a comparison of the levels of transcripts of all known ETS and GATA factors between MS1, H5V, EOMA, and 1G11 endothelial cells and L929 (Figure S7) and 3T3 fibroblasts (not shown) was performed. Among the six GATA factors analyzed, GATA-2 was, by far, the most highly expressed in endothelial cells when compared to L929, with endothelial/fibroblast ratios ranging from 22±8.5 (EOMA) to 57.4±2.0 (H5V). Regarding the ETS family, endothelial cells were mostly enriched in Erg and Fli-1 when compared to fibroblasts, with endothelial/fibroblast ratios ranging from 692.2±0.0 to 909.0±0.0 for Erg and 26.9±0.0 to 102.2±0.6 for Fli-1, whereas most other factors were expressed at levels close to those observed in fibroblasts.

**Figure 7:**
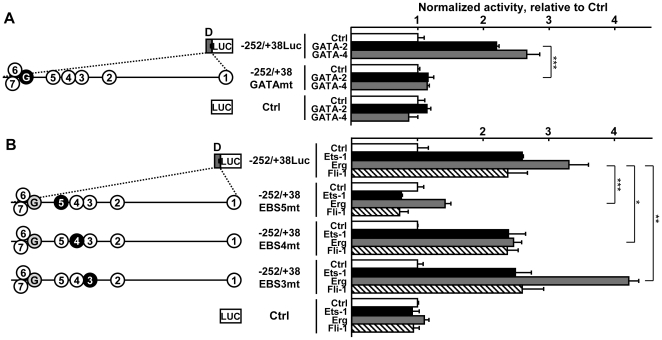
Region D is transactivated by ETS and GATA factors. **A**. Right; L929 cells were transfected with 70 fmoles of pcDNA3 expression vector (Ctrl, white bars) or with pcDNA3 constructs expressing the mouse GATA-2 (black bars) or GATA-4 (grey bars) and with the indicated Luc reporter vectors (80 fmoles), together with 54 fmoles of pCH110 normalization vector. After 48h of culture, cells were lyzed and the luciferase value of each sample was measured and normalized with its β-galactosidase value. Bars represent normalized activity as fold over the Ctrl mean value of either Luc reporter set to 1. Left; scaled schematic representation of region D. The location of the GATA (circled G) and EBS (circled numbers) sites is represented to scale along the schematized region D. Mutated sites are represented as blackened circles. Names of the constructs are given according to the cloned 5′ and 3′ end positions relative to the exon-1b transcription start, LUC; luciferase. GATA-2 and GATA-4 transactivated the wild-type −252/+38 promoter region while mutation of the GATA site abolished the response of the promoter to these GATA factors. **B**. Right; L929 cells were transfected with 70 fmoles of pcDNA3 expression vector (Ctrl, white bars) or with pcDNA3 constructs expressing the mouse Ets-1 (black bars), Erg (grey bars) or Fli-1 (dashed bars) and with 80 fmoles of the indicated Luc reporter vectors together with 54 fmoles of pCH110 normalization vector. After 48h of culture, cells were lyzed and the luciferase value of each sample was measured and normalized with its β-galactosidase value. Bars represent normalized activity as fold over the Ctrl mean value of either Luc reporter set to 1. Left; scaled schematic representation of region D. The location of the GATA (circled G) and EBS (circled numbers) sites is represented to scale along the schematized region D. Mutated sites are represented as blackened circles. Names of the constructs are given according to the cloned 5′ and 3′ end positions relative to the exon-1b transcription start, Luc; luciferase. The −252/+38 promoter region responds to Ets-1, Erg and Fli-1 and mutation of EBS5 abolished the response to the ETS factors. Mutation of either EBS3 or of EBS4 had no effect on the response to Ets-1 or to Fli-1, however, mutation of EBS3 increased the response to Erg, that of EBS4 decreased it, *** p<0.001, ** p<0.01, * p<0.05.

We then transfected L929 cells with the −252/+38Luc reporter or mutated versions of it together with expression vectors coding for either GATA-2, GATA-4, Ets-1, Erg, or Fli-1 in order to assess which of these transcription factors would transactivate this promoter region. GATA-2 and GATA-4 transactivated the −252/+38Luc construct (Figure 7A) while mutation of the GATA site abolished the transactivation induced by both factors. In similar assays, Erg, Fli-1, and Ets-1 transactivated the −252/+38Luc reporter (Figure 7B) and mutation of EBS5 abolished the response to Fli-1 and to Ets-1 and decreased the response to Erg by 86% when compared to Ctrl. Mutation of EBS4 did not significantly affect the response of the promoter to Fli-1 or to Ets-1 and slightly decreased the response to Erg. Mutation of EBS3 had no effects on the response to Fli-1 or to Ets-1 and increased the response to Erg.

Thus, when over-expressed, Erg, Fli-1 and Ets-1 were able to transactivate the proximal promoter region mainly through EBS5. On the other hand, Erg, Fli-1 and Ets-1 cannot account for the effects observed with the EBS3mt construct in Figure 6B, suggesting that other ETS factors regulate the expression of the gene through this site.

### Endogenous Erg and GATA-2 regulate the expression of *VE-statin/egfl7* in endothelial cells

**Figure 8:**
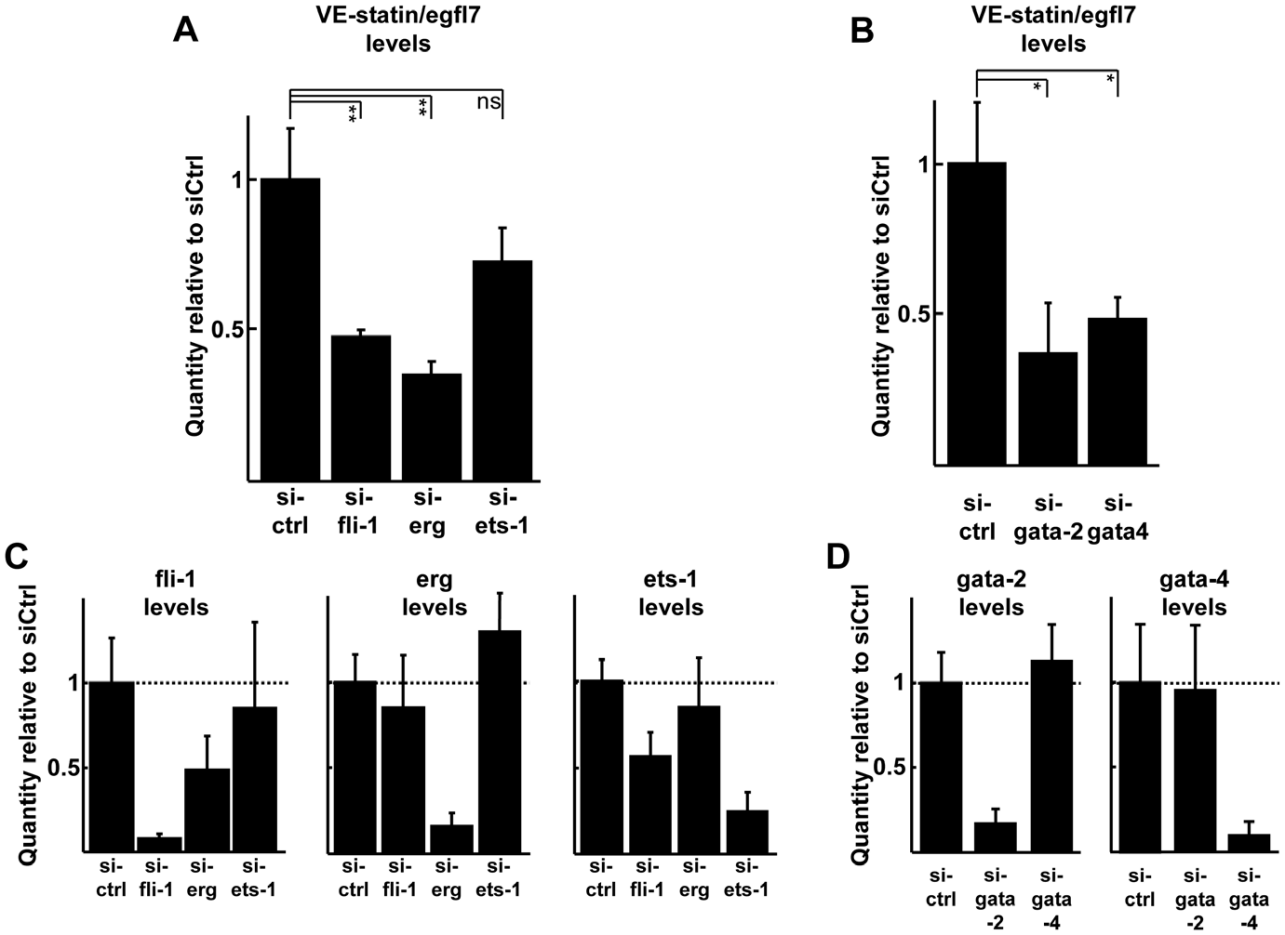
Down-regulation of endogenous ETS and GATA factors in endothelial cells. **A**. H5V endothelial cells were transfected with siRNA targeting fli-1, erg, or ets-1 (**A**), gata-2, or gata-4 (**B**) and cultured 48h before total RNA extraction and qPCR analysis of endogenous *VE-statin/egfl7* expression. ** p<0.01, ns; not significant. Quantities are relative to mean value of the Ctrl siRNA set to 1. Down-regulation of Erg, Fli-1, gata-2 and gata-4 resulted in a significant decrease in *VE-statin/egfl7* expression levels. Specificity of the siRNA: qPCR analysis of expression levels of the *erg, fli-1* and *ets-1* (**C**) and of *gata-2* and *gata-4* (**D**) genes in response to either siRNA. Levels were normalized to the mean value obtained in response to transfection to the Ctrl siRNA set to 1. All siRNA show a good specificity of inhibition toward their target and no significant side effects on the other studied genes of the same family. Of note, the siRNA targeting Ets-1 down-regulates Fli-1 as well, as expected since Ets-1 controls the *fli-1* gene in endothelial cells [66].

In order to address the role of the endogenous transcription factors on the regulation of the *VE-statin/egfl7* gene in endothelial cells, Erg, Fli-1, Ets-1, GATA-2 and GATA-4 were targeted using RNA interference. siRNA-mediated down-regulation of the fli-1 transcripts (92%, Figure 8C) and that of erg (84%, Figure 8C) induced a 53% and 65% drop of VE-statin/egfl7 levels in endothelial cells, respectively (Figure 8A). On the other hand, down-regulation of ets-1 (75%, Figure 8C) did not significantly affect VE-statin/egfl7 expression. Down-regulation of GATA-2 (83%, Figure 8D) or of GATA-4 (90%, Figure 8D) induced a 63% and 55% drop of *VE-statin/egfl7* gene expression in endothelial cells, respectively (Figure 8B).

**Figure 9:**
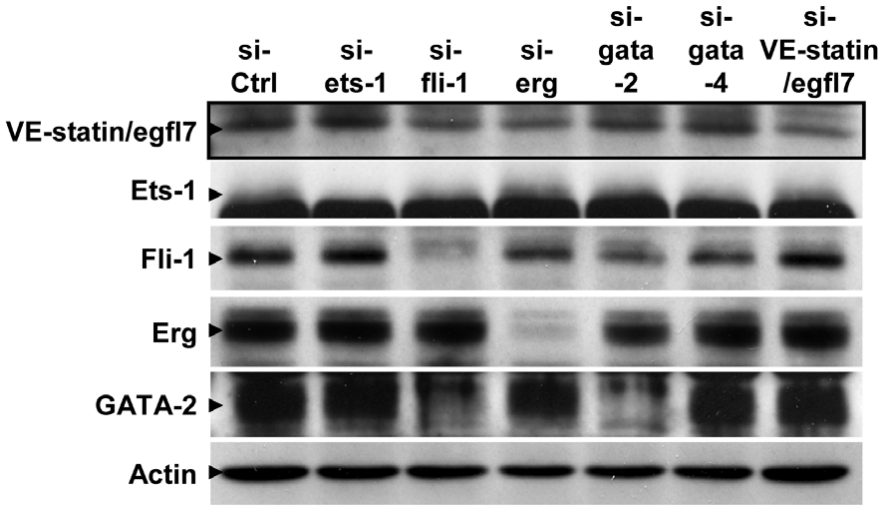
Targeting of Fli-1, Erg and GATA-2 affects VE-statin/egfl7 protein accumulation in endothelial cells. H5V endothelial cells were transfected with a control siRNA (siCtrl) of with siRNA targeting ets-1, fli-1, erg, gata-2, or gata-4, cultured for 48hr and analysed for protein expression by SDS-PAGE and Western-blotting. VE-statin/egfl7 accumulation (boxed) is impaired when cells were treated with siRNA targeting fli-1, erg and, and, to a slight extent, GATA-2, or when treated with a siRNA targeting VE-statin/egfl7 itself, used as positive control. Of note, there is a cross-regulation between gata-2 and fli-1, as already reported in hematopoietic stem cells [37]. Specific bands are indicated by arrowheads. The Ets-1 band is distinguishable from a large non-specific band located immediately below [67].

These results were confirmed at the protein levels by Western-blot analyses which showed that VE-statin/egfl7 accumulation was impaired in cells treated with a siRNA targeting fli-1, erg, or, to a slight extent, GATA-2, but not those targeting GATA-4 or Ets-1 (Figure 9). Interestingly, targeting fli-1 induced the repression of GATA-2 (in addition to fli-1) and vice-versa, confirming previous observations on this cross-regulation [37].

**Figure 10:**
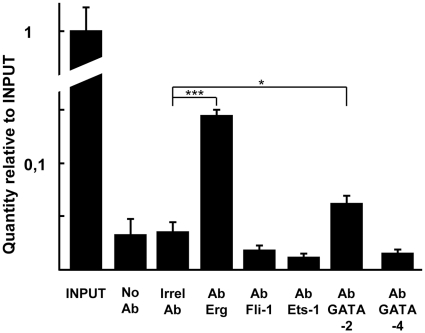
Endogenous Erg and GATA-2 are bound to the −252/+38 region of the *VE-statin/egfl7* promoter. H5V cell cultures were treated with 1% formaldehyde, sonicated in order to produce 1000 bp average size DNA fragments and immunoprecipitation of the DNA-protein complexes was performed using antibodies against HA.11 (irrelevant Ab, MMS-101R, Convance), Erg (sc-354x, Santa-Cruz), Fli-1 (sc-356x, Santa-Cruz), Ets-1 (sc-111x, Santa Cruz), GATA-2 (sc-9008x, Santa-Cruz), or GATA-4 (sc-1237x, Santa-Cruz) antibody. DNA was then purified and analyzed by semi-quantitative PCR on the −252/+38 promoter region of the endogenous *VE-statin/egfl7* gene. INPUT; 3.5% of total DNA, No Ab; no antibody added, Irrel Ab; irrelevant IgG added. Levels were measured by semi-quantitative PCR using oligonucleotides that span the −252/+38 region of the gene. Data are expressed as 2^−ΔCt^ where ΔCt = Ct of sample – Ct of INPUT. The experiment is representative of a set of three performed in similar conditions. *** p<0.001, * p<0.05.

Finally, chromatin immunoprecipitation assays using endothelial cell extracts showed that Erg and GATA-2 are present onto the −252/+38 region of the *VE-statin/egfl7* promoter in endothelial cells (Figure 10) whereas Fli-1, Ets-1 and GATA-4 could not be detected on this region, strongly suggesting that Erg and GATA-2 directly regulate the proximal region of the *VE-statin/egfl7* gene while Fli-1 would have an indirect regulatory role, possibly through the regulation of GATA-2.

## Discussion

The present work shows that the *VE-statin/egfl7* gene is not located in a large endothelial-specific cluster on chromosome 2. Rather, its specific expression in endothelial cells depends on epigenetic mechanisms and on the presence of an enhancer located 8.4 kb ahead of the main transcription start site which provides strong levels of expression in endothelial cells. The proximal −252/+38 promoter region also participates to the endothelial-specificity of expression of the gene, in particular through essential ETS and GATA binding sites. This proximal region is occupied by the Erg and GATA-2 transcription factors which are both involved in the expression of the endogenous gene in endothelial cells.

Region A, acting as an enhancer, was the most active region identified by deletion analysis *in vitro*. However, despite a thorough and frustrating analysis, we were not able to attribute a prominent role to any discrete site or sub-region of this enhancer in the regulation of expression of the gene, a situation similar to the endothelial REn1 enhancer of the *robo4* promoter [8]. Our data altogether show that this region as a whole carries important regulatory features. It may be involved in DNA-loop interactions that would bring it in close proximity to the transcription start site of the gene and favor transcription in endothelial cells, such as reported for *prolactin* [38] or *prostate specific antigen* [39] but, so far, we have no experimental data that could support this hypothesis.

Most of the information gathered here showed the importance of the −252/+38 region of the promoter. This region is necessary for expression of the gene and is affected by epigenetic modifications that increase the chromatin accessibility specifically in endothelial cells. Among the conserved putative binding sites that are present in this region, two EBS and one GATA are essential for activity. Mutation of the GATA site in region D resulted in the loss of half of the activity in endothelial cells, underlining the important role of this site. GATA factors have been frequently associated with endothelial gene expression; important GATA sites are present in the promoters or enhancers of genes like *flk-1* [2], *VE-cadherin* [5], *endothelin-1* [40], *eNOS* [41], *icam-2* [42], or *vWF* [43] and the presence of a GATA site has been predicted in a putative endothelial and hematopoietic promoter signature in a genome-wide analysis [44]. Our data confirm that among the different GATA transcription factors, *gata-2* is markedly expressed in endothelial cells [45]. Interestingly, the active GATA site in the *VE-statin/egfl7* promoter matches the canonical WGATAR sequence that allows high expression of *gata-2* itself in endothelial cells [46], suggesting that a similar regulation could control *VE-statin/egfl7* expression in these cells.

The involvement of EBS in the regulation of genes in endothelial cells is also well documented. Such sites play essential roles in the regulation of expression of *flk-1*, *tie-1*, *tie-2*, *robo-4* or *VE-cadherin* in endothelial cells [14], [47]. Among the EBS analyzed, the individual mutation of EBS3 or of EBS5 markedly affected the activity of the reporter in endothelial and in fibroblast cells, but with a greater effect with EBS5. This suggests that these two sites are necessary both for basal expression (in any cell type) and for specific expression in endothelial cells. It was particularly striking to see that the mutation of two bases of the EBS5 was sufficient to abolish the activity of the long and most active −8409/+38Luc reporter. Such dramatic effect pinpoints the crucial importance of this site for expression of the gene.

Regarding the other EBS, mutation of EBS6 and that of EBS1 induced an interesting doubling of the endothelial/fibroblast ratio when compared to −8409/+38Luc, mostly due to a decrease of expression in fibroblasts. This probably explains the fact that the scanD-7 mutant presented both a large drop in activity, which is correlated to the loss of the GATA site, and a raise of the endothelial/fibroblast ratio due to the mutation of EBS6. Mutation of EBS2 lowered the endothelial/fibroblast ratio probably because of a raise of activity in fibroblasts, suggesting that an ETS repressor acts on this site in non-endothelial cells in order to refrain expression. The ETS family contains transcriptional repressors, such as Tel, Net and Erf among which Tel is widely expressed in endothelial cells and participates to the maintenance of the developing vascular network [48]. Interestingly, *Tel* expression levels were 2.9 to 6.4-times higher in L929 fibroblasts than in endothelial cells, suggesting that this factor could act as a repressor of *VE-statin/egfl7* expression in non-endothelial cells. However, we have yet no evidence that Tel would affect *VE-statin/egfl7* expression.

The levels of ETS factors in endothelial cells observed here are in accordance with those measured earlier in several cell lines and tissues in an expression profile analysis [49]. In this study, Erg was, by far, the most highly expressed ETS factor in human endothelial cells, followed by a group that included Fli-1, Sap1, Net, GAPBα and Ets-2. Among the ETS factors that may regulate the EBS of the *VE-statin/egfl7* promoter, Erg and Fli-1 thus logically emerged as potential candidates in regard to their very high expression in endothelial cells versus fibroblasts (Figure S7B and [49]). In addition, Erg and Fli-1 are co-expressed in the endothelium during development [50], [51] and act together and additively in order to regulate angiogenesis in zebrafish, possibly through a co-regulation of *VE-cadherin* [52]. The Erg transcription factor regulates the *VE-cadherin* [53], and the *endoglin* promoters and enhancer together with Fli-1 and Elf-1 [54]. It should be however noted that Erg loss-of-function does not induce an overt vascular phenotype in homozygous mutant mice, as blood vessels seem normal, even though the primitive vascular network shows dilated vessels [55]. On the other hand, it was quite surprising to see that Ets-1 was not more highly expressed in endothelial cells than in fibroblasts. Ets-1 is expressed in endothelial cells during development [56] and has repeatedly been associated with endothelial gene expression when used in over-expression approaches, including that of *flk-1*, *tie* and *VE-cadherin* [57]. However, the various *ets-1* gene inactivation approaches that were reported did not relate any vascular defects that would give Ets-1 a major role in the endothelial fate [58], [59], [60]. On the other hand, a gene co-inactivation approach recently showed that Ets-1 and Ets-2 are together involved in vessel branching and endothelial survival [61]. This illustrates the importance of redundancy of action between ETS factors for specific gene expression, including that of Ets-1 in endothelial cells.

Mutation of EBS3 induced a marked decrease in promoter activity while this mutant still responded to Erg, Fli-1 and Ets-1 in transactivation assays, suggesting that other ETS factors are involved in the regulation of the proximal *VE-statin/egfl7* promoter in endothelial cells. Few other ETS factors have a documented role in vasculogenesis or angiogenesis; the *etv2/EtsRP71* gene inactivation prevents the emergence of the endothelial lineage and the angiopoietin receptor *tie-2* is a direct target gene of this transcription factor [62]. Similarly, *elk-3/net/sap-2* inactivation induces dilated lymphatics [63] and *etv-6/tel* inactivation induces a lack of vitelline vessels [48]. The fact that neither Etv2/EstRP71, Elk-3/Net/Sap-2 nor Etv-6/Tel were up-regulated here in endothelial cells does not exclude the possibility that these ETS factors regulate *VE-statin/egfl7* expression in endothelial cells without being prominently expressed.

Interestingly, a RYMAAYA consensus sequence is present in region D of the mouse *VE-statin/egfl7* at −207/−212, in tandem with a putative EBS. This sequence corresponds to the proposed Forkhead sequence that, when combined with an EBS, is suggested to be important for the regulation of genes in endothelial cells [13], [14]. However, this sequence is present at this location only in the mouse *VE-statin/egfl7* sequence, it is not conserved among species, including rat and human (Figure S6). Second, it is located in the sequences corresponding to scanD-2 and scanD-3 sequences which global activity and endothelial/fibroblast ratio were not affected by mutation (Figure 5A). It is therefore unlikely that this putative Forkhead/EBS in region D plays an important role in the regulation of the *VE-statin/egfl7* gene in endothelial cells. This raises the importance of experimentally validating such site prediction.

It was recently observed that the microRNA miR-126 located in the seventh intron of the *VE-statin/egfl7* gene is specifically expressed in endothelial cells [64]. Although it is not yet demonstrated, it is probable that this microRNA arises from the VE-statin/egfl7 pre-mRNA and depends on the same regulation mechanisms. A briefly described analysis showed that miR-126 expression is dependent on the presence of two regions containing ETS binding sites, one of which responds to the over-expression of Ets-1 [64]. Interestingly, a sequence located in the present region D and which contains EBS5 and EBS4 provided an endothelial-specific pattern of expression [64]. This observation correlates well with our results here showing the importance of this region for expression of the gene in endothelial cells. However, since tissue-specific co-regulation of a microRNA and of its host gene are not strictly linked [65], whether the expression of *VE-statin/egfl7* and that of miR-126 are regulated by similar mechanisms in endothelial cells remains to be investigated.

## Supporting Information

Figure S1 Histone modifications along the 5′ region of the *VE-statin/egfl7* gene A. Levels of acetylated histone H4 along the 5′ region of the *VE-statin/egfl7* gene. H5V endothelial cells (•, black bars) and L929 (□, white bars) fibroblast cells were processed for chromatin immunoprecipitation as described in Figure 2 using an anti-acetyl-histone H4 antibody (06-866, Millipore). Inset: Acetylated Histone H4 levels of the negative control *β-globin* and the positive control *VE-cadherin* gene promoters taken as non- and highly-expressed genes in endothelial cells, respectively. Semi-quantitative PCR were performed in order to amplify various locations along the promoter represented as the x-axis. Quantities are relative to the diluted INPUT mean value set to 1.
(0.15 MB TIF)

Figure S2 Na butyrate releases expression of *VE-statin/egfl7* in non-endothelial cells HeLa cells (white bars) and H5V (black bars) were treated with 2 mM Na butyrate for 72 hr, lysed, total RNA isolated and VE-statin/egfl7 mRNA levels quantified by qPCR (see Material and Methods). Values are normalized to GAPDH levels of the corresponding samples, levels of untreated cells are set to 1.
(0.04 MB TIF)

Figure S3 Identification of conserved regions between the mouse and human *VE-statin/egfl7* promoters. 20kb of mouse and human *VE-statin/egfl7* gene promoter sequences located upstream of exon-3 were aligned using BLASTN v2.2.6 (www.ncbi.nlm.nih.gov/blast/). Sequences with more than 80% identity located in corresponding positions and in the same strand direction were used to define the conserved A to E regions. The identified conserved regions are represented to scale on the promoter region as lettered gray boxes, the exons as numbered black boxes, the two transcription starts are represented as arrows.
(0.42 MB TIF)

Figure S4 The mouse promoter is functional in human primary endothelial cells. Right: Human primary endothelial HUVEC cells (black bars) were transfected with pGL3basic (Ctrl) or pGL3basic in which the −12969/+38 *VE-statin/egfl7* promoter region or 5′ deletions of it were inserted. These reporters (80 fmoles) were transfected together with 54 fmoles pCMV-βGal normalization vector. After 48h of culture, cells were lyzed and the luciferase value of each sample was measured and normalized with its β-galactosidase value. Bars represent normalized activity as fold over Ctrl mean value set to 1. Left: scaled schematic representation of the constructs, names are given according to the cloned 5′ and 3′ end positions relative to the exon-1b transcription start, Luc; luciferase. The experiment is representative of a set of two experiments performed in similar conditions, ** p<0.01.
(0.06 MB TIF)

Figure S5 Linker-scanning analysis of the −8409/−7530 region. H5V endothelial cells (black bars) and L929 fibroblasts (white bars) were transfected with 80 fmoles of pGL3basic (Ctrl), −8409/+38Luc or mutated versions of this vector (scan A-1 to -6) corresponding to the successive exchange of 20bp of wild-type for a 20bp transactivation-null cassette in the −7770/−7570 region and with 54 fmoles of pCH110 normalization vector. After 48h of culture, cells were lyzed and the luciferase value of each sample was measured and normalized with its β-galactosidase value. Bars represent normalized activity as fold over Ctrl mean value set to 1. Results are displayed as in Figure 3A. The experiment is representative of sets of three experiments performed in similar conditions. No sub-region was reliably found to be important for activity of this sequence.
(0.07 MB TIF)

Figure S6 Conservation analysis of the *VE-statin/egfl7* region D in vertebrates. Pairwise alignments of vertebrate genomic sequences were realized using the comparative genomics tool (30-Way Multiz Alignment & Conservation) of the UCSC/Penn State genome browser utilities (http://genome.ucsc.edu) with the mouse *VE-statin/egfl7* gene as template. Conserved putative binding sites are boxed, bases matching the expected consensus are bolded.
(0.77 MB TIF)

Figure S7 A. Expression levels of GATA transcription factors were measured by RT-qPCR using total RNA isolated from L929 fibroblasts and from 1G11, H5V, EOMA, and MS1 endothelial cells. Levels are normalized to GAPDH quantities and are represented as folds over the levels measured in L929 cells, using the ΔΔCt method. Y-axis scales are Log10 representations. The oligonucleotide pairs used for each transcript and the qPCR conditions are described in Table S3 and in the Material and Methods sections, respectively. B. Expression levels of ETS transcription factors were measured using qPCR in cDNA isolated from L929 fibroblasts and from 1G11, H5V, EOMA, and MS1 endothelial cells. Levels are normalized to GAPDH quantities and are represented as folds over the levels measured in L929 cells, using the ΔΔCt method. Y-axis scales are Log10 representations. The oligonucleotide pairs used for each transcript and the qPCR conditions are described in Table S3 and in the Materials and Methods sections, respectively.
(0.28 MB TIF)

Table S1 Primers used for site-directed mutagenesis and linker scanning analysis. Primers are listed in the 5′→3′ orientation.
(0.07 MB DOC)

Table S2 Primers and conditions used in qPCR analysis following ChIP. Primers are listed in the 5′→3′ orientation.
(0.03 MB DOC)

Table S3 Primers used in qPCR. Primers are listed in the 5′→3′ orientation.
(0.05 MB DOC)
