# Supplementary figures and images for: VE-statin/egfl7 Expression in Endothelial Cells Is Regulated by a Distal Enhancer and a Proximal Promoter under the Direct Control of Erg and GATA-2
Source: PLoS One. 2010 Aug 16;5(8):e12156. doi: 10.1371/journal.pone.0012156 (PMC2922337; doi:10.1371/journal.pone.0012156)

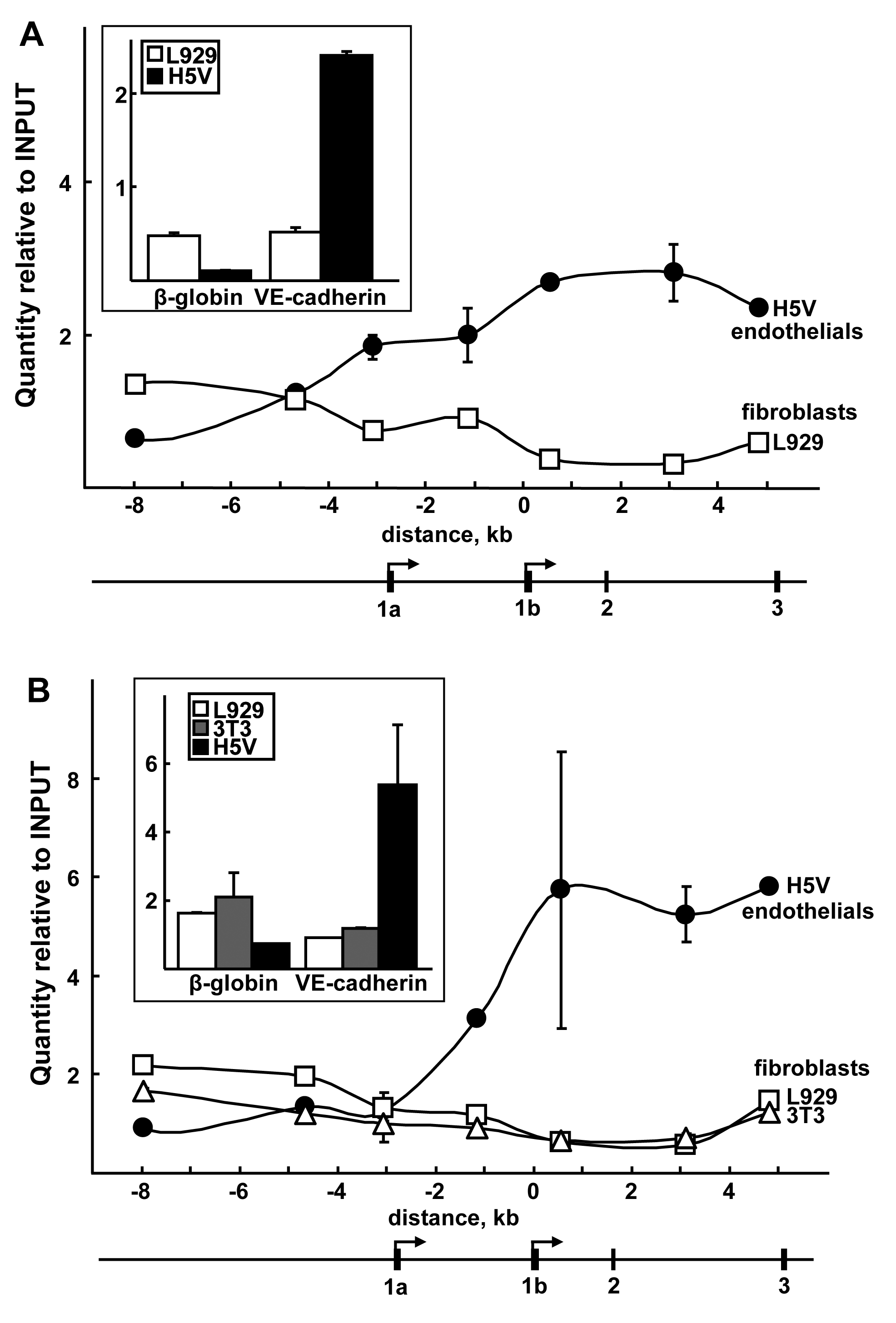

Supplement: Figure S1 — Histone modifications along the 5′ region of the VE-statin/egfl7 gene A. Levels of acetylated histone H4 along the 5′ region of the VE-statin/egfl7 gene. H5V endothelial cells (•, black bars) and L929 (□, white bars) fibroblast cells were processed for chromatin immunoprecipitation as described in Figure 2 using an anti-acetyl-histone H4 antibody (06-866, Millipore). Inset: Acetylated Histone H4 levels of the negative control β-globin and the positive control VE-cadherin gene promoters taken as non- and highly-expressed genes in endothelial cells, respectively. Semi-quantitative PCR were performed in order to amplify various locations along the promoter represented as the x-axis. Quantities are relative to the diluted INPUT mean value set to 1. (0.15 MB TIF) [file pone.0012156.s001.tif]

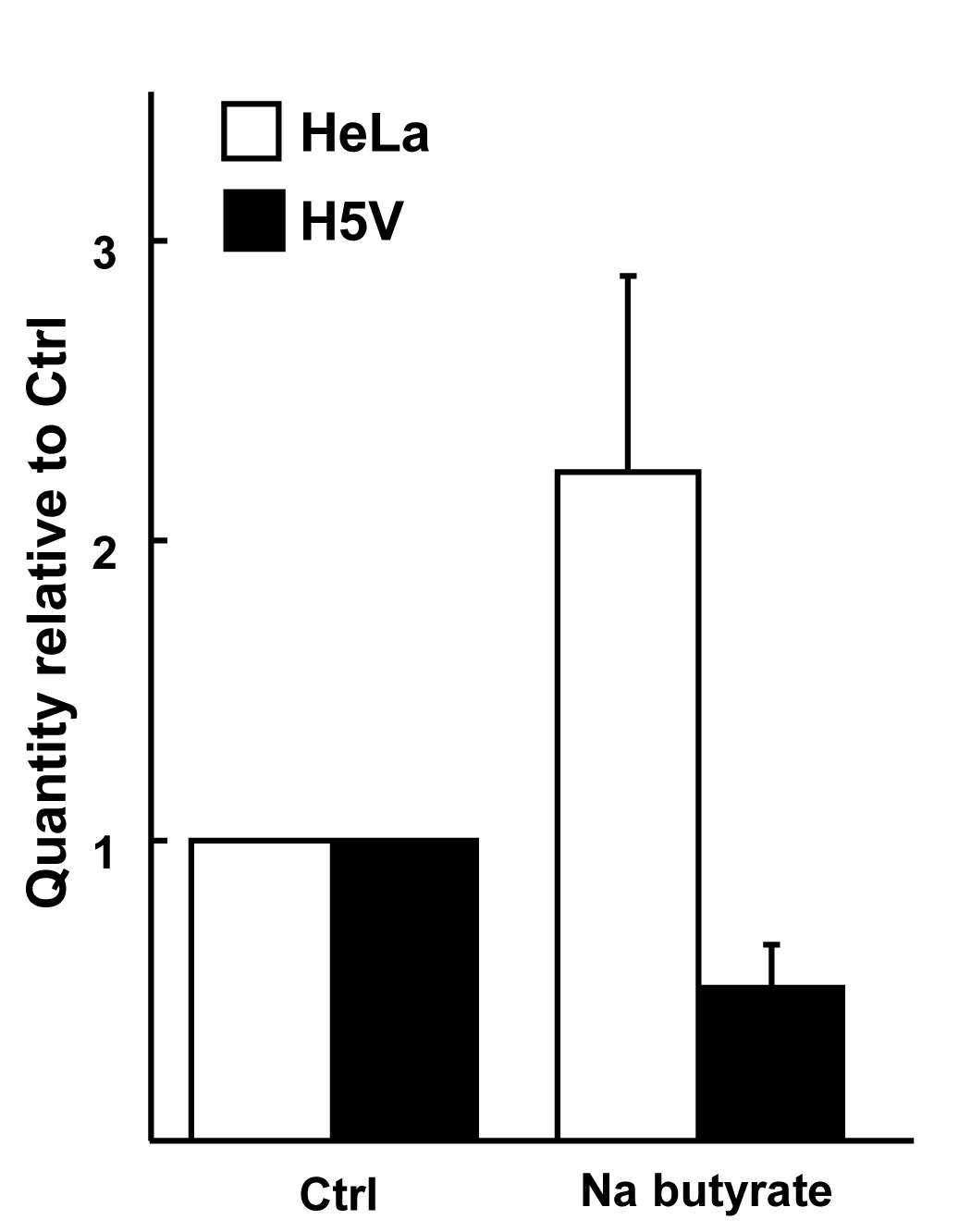

Supplement: Figure S2 — Na butyrate releases expression of VE-statin/egfl7 in non-endothelial cells HeLa cells (white bars) and H5V (black bars) were treated with 2 mM Na butyrate for 72 hr, lysed, total RNA isolated and VE-statin/egfl7 mRNA levels quantified by qPCR (see Material and Methods). Values are normalized to GAPDH levels of the corresponding samples, levels of untreated cells are set to 1. (0.04 MB TIF) [file pone.0012156.s002.tif]

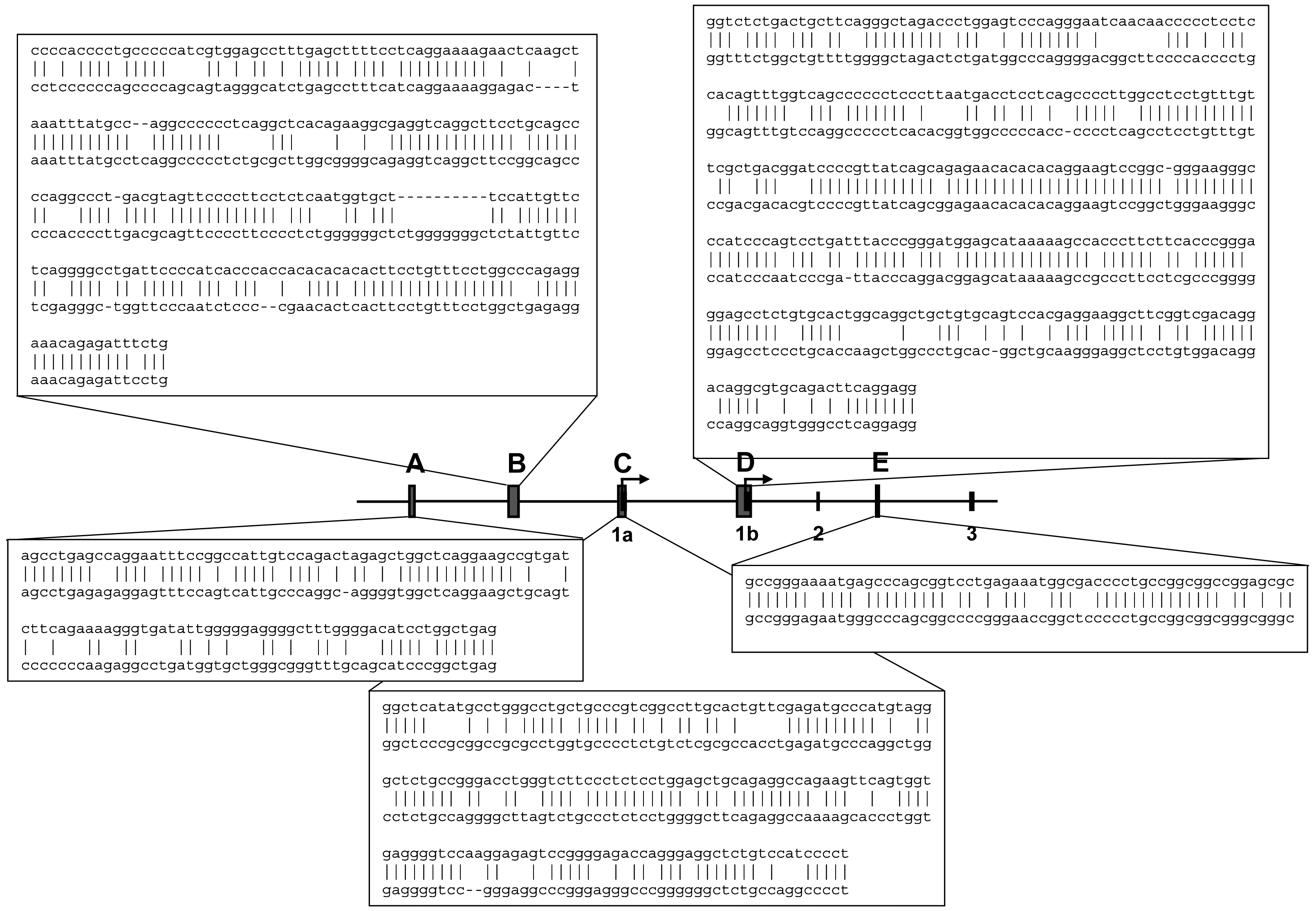

Supplement: Figure S3 — Identification of conserved regions between the mouse and human VE-statin/egfl7 promoters. 20kb of mouse and human VE-statin/egfl7 gene promoter sequences located upstream of exon-3 were aligned using BLASTN v2.2.6 (www.ncbi.nlm.nih.gov/blast/). Sequences with more than 80% identity located in corresponding positions and in the same strand direction were used to define the conserved A to E regions. The identified conserved regions are represented to scale on the promoter region as lettered gray boxes, the exons as numbered black boxes, the two transcription starts are represented as arrows. (0.42 MB TIF) [file pone.0012156.s003.tif]

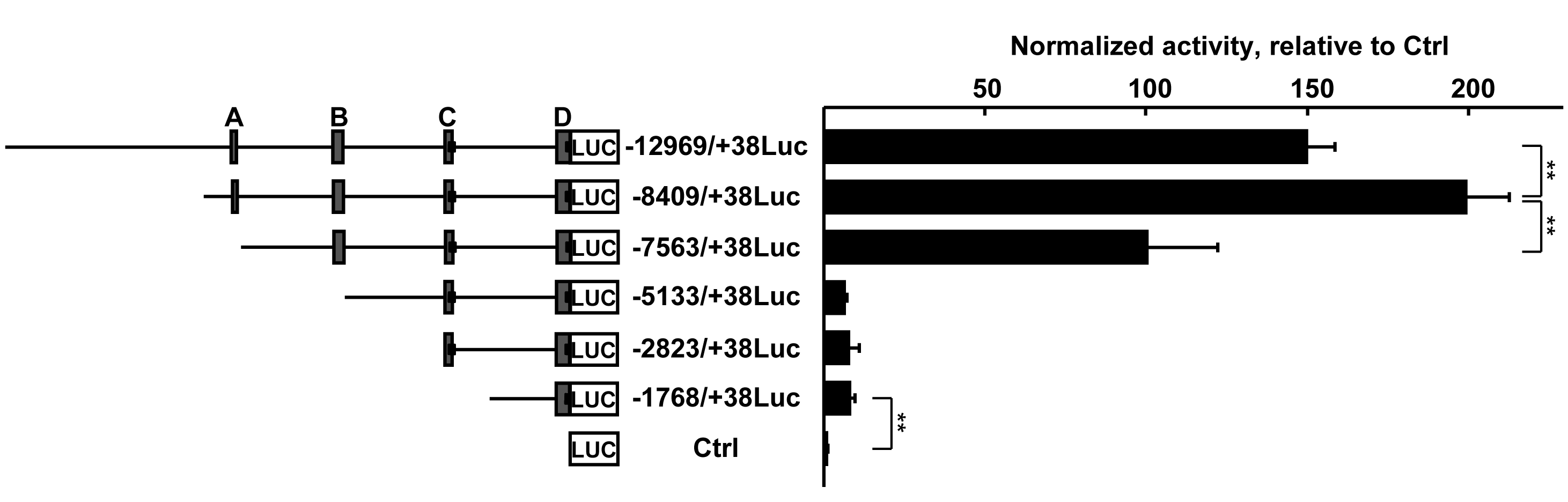

Supplement: Figure S4 — The mouse promoter is functional in human primary endothelial cells. Right: Human primary endothelial HUVEC cells (black bars) were transfected with pGL3basic (Ctrl) or pGL3basic in which the −12969/+38 VE-statin/egfl7 promoter region or 5′ deletions of it were inserted. These reporters (80 fmoles) were transfected together with 54 fmoles pCMV-βGal normalization vector. After 48h of culture, cells were lyzed and the luciferase value of each sample was measured and normalized with its β-galactosidase value. Bars represent normalized activity as fold over Ctrl mean value set to 1. Left: scaled schematic representation of the constructs, names are given according to the cloned 5′ and 3′ end positions relative to the exon-1b transcription start, Luc; luciferase. The experiment is representative of a set of two experiments performed in similar conditions, ** p<0.01. (0.06 MB TIF) [file pone.0012156.s004.tif]

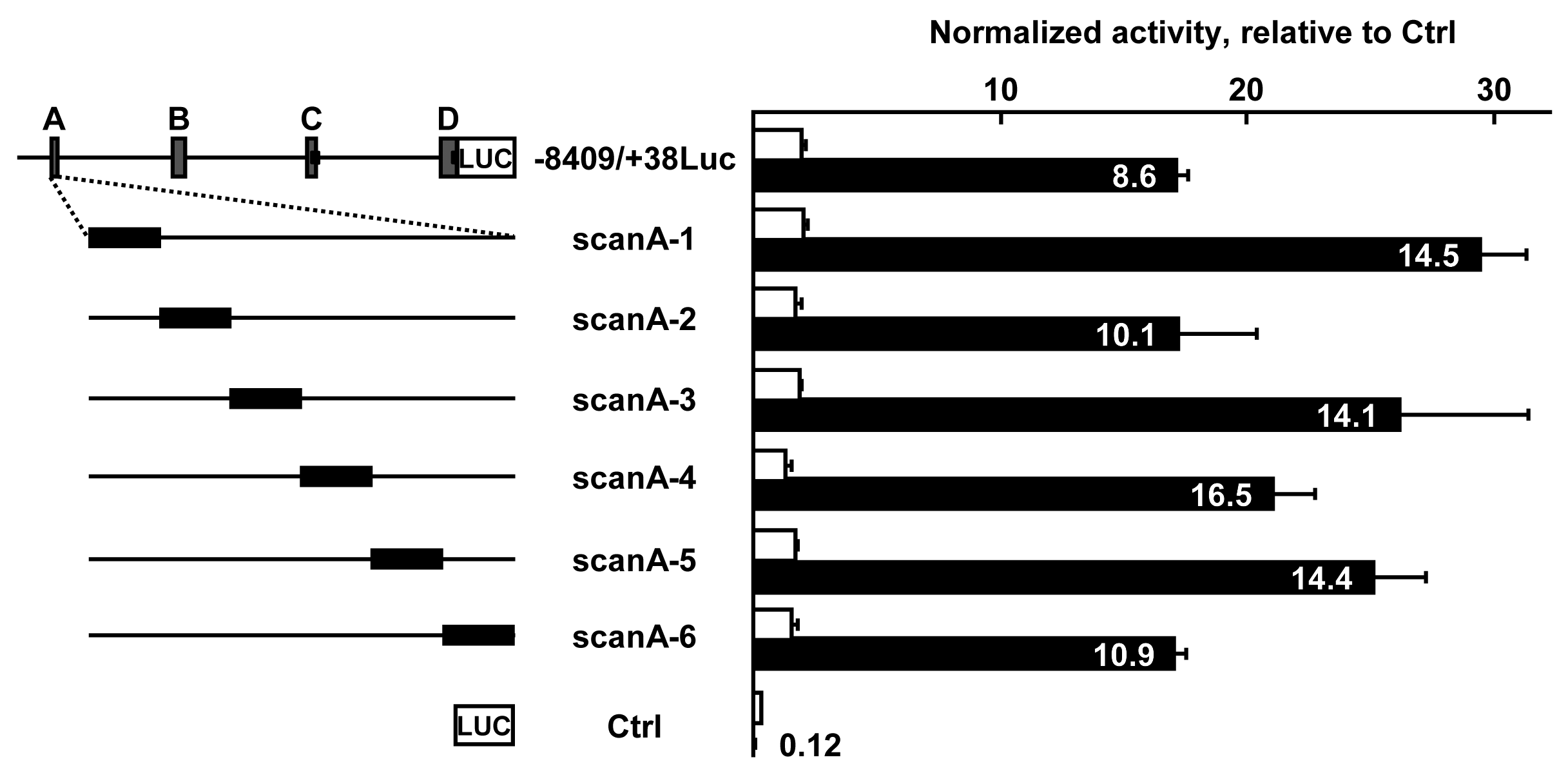

Supplement: Figure S5 — Linker-scanning analysis of the −8409/−7530 region. H5V endothelial cells (black bars) and L929 fibroblasts (white bars) were transfected with 80 fmoles of pGL3basic (Ctrl), −8409/+38Luc or mutated versions of this vector (scan A-1 to -6) corresponding to the successive exchange of 20bp of wild-type for a 20bp transactivation-null cassette in the −7770/−7570 region and with 54 fmoles of pCH110 normalization vector. After 48h of culture, cells were lyzed and the luciferase value of each sample was measured and normalized with its β-galactosidase value. Bars represent normalized activity as fold over Ctrl mean value set to 1. Results are displayed as in Figure 3A. The experiment is representative of sets of three experiments performed in similar conditions. No sub-region was reliably found to be important for activity of this sequence. (0.07 MB TIF) [file pone.0012156.s005.tif]

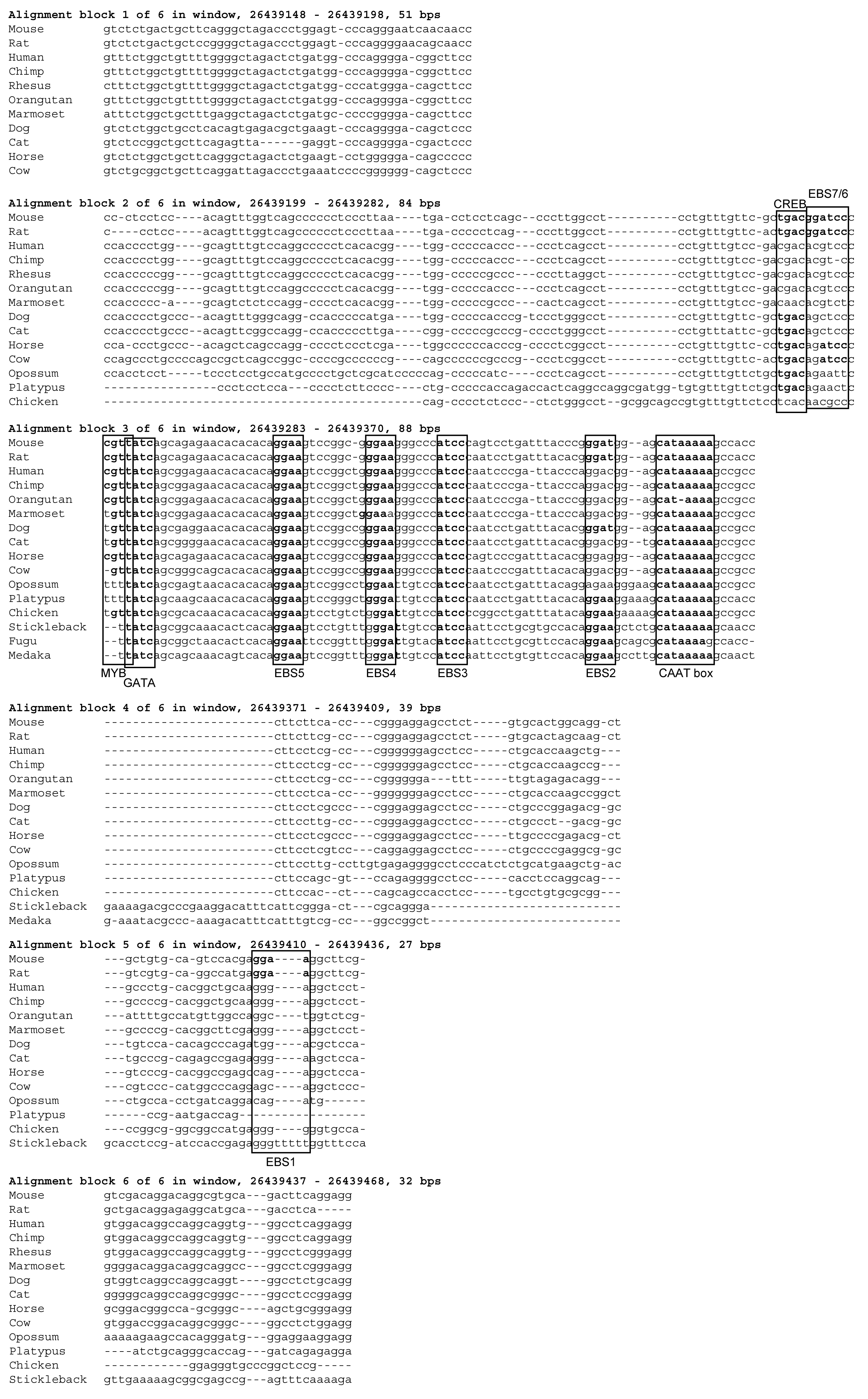

Supplement: Figure S6 — Conservation analysis of the VE-statin/egfl7 region D in vertebrates. Pairwise alignments of vertebrate genomic sequences were realized using the comparative genomics tool (30-Way Multiz Alignment & Conservation) of the UCSC/Penn State genome browser utilities (http://genome.ucsc.edu) with the mouse VE-statin/egfl7 gene as template. Conserved putative binding sites are boxed, bases matching the expected consensus are bolded. (0.77 MB TIF) [file pone.0012156.s006.tif]

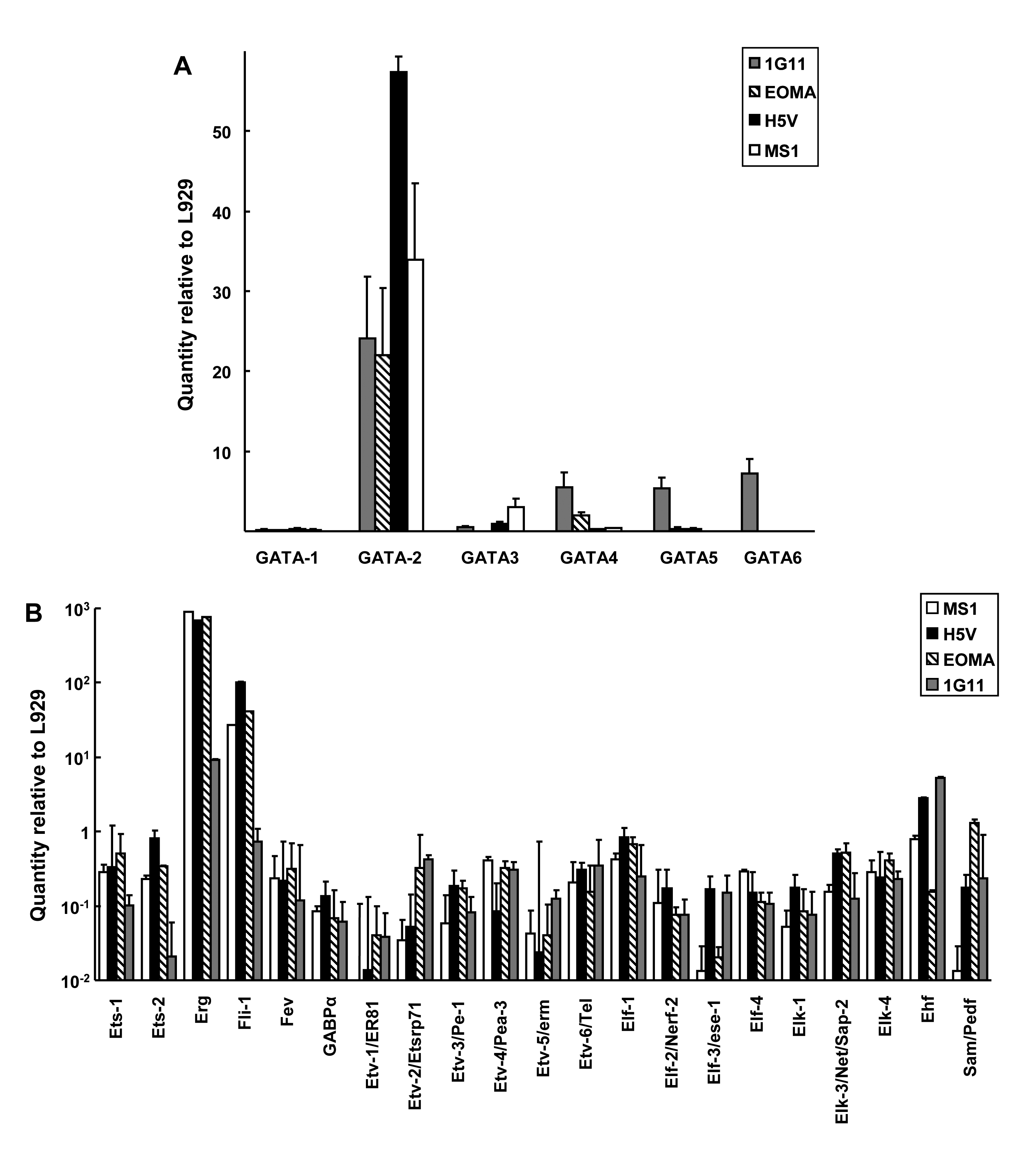

Supplement: Figure S7 — A. Expression levels of GATA transcription factors were measured by RT-qPCR using total RNA isolated from L929 fibroblasts and from 1G11, H5V, EOMA, and MS1 endothelial cells. Levels are normalized to GAPDH quantities and are represented as folds over the levels measured in L929 cells, using the ΔΔCt method. Y-axis scales are Log10 representations. The oligonucleotide pairs used for each transcript and the qPCR conditions are described in Table S3 and in the Material and Methods sections, respectively. B. Expression levels of ETS transcription factors were measured using qPCR in cDNA isolated from L929 fibroblasts and from 1G11, H5V, EOMA, and MS1 endothelial cells. Levels are normalized to GAPDH quantities and are represented as folds over the levels measured in L929 cells, using the ΔΔCt method. Y-axis scales are Log10 representations. The oligonucleotide pairs used for each transcript and the qPCR conditions are described in Table S3 and in the Materials and Methods sections, respectively. (0.28 MB TIF) [file pone.0012156.s007.tif]
