# Supplementary material for: VE-statin/egfl7 Expression in Endothelial Cells Is Regulated by a Distal Enhancer and a Proximal Promoter under the Direct Control of Erg and GATA-2
Source: PLoS One. 2010 Aug 16;5(8):e12156. doi: 10.1371/journal.pone.0012156 (PMC2922337; doi:10.1371/journal.pone.0012156)
Supplement: Table S1 — Primers used for site-directed mutagenesis and linker scanning analysis. Primers are listed in the 5′→3′ orientation. (0.07 MB DOC) [file pone.0012156.s008.doc]

**Site-directed mutagenesis**

| **Construct** | **foward primer** | **reverse primer** |
| --- | --- | --- |
| -8409/+38GATAmt | cccgtttttagcagagaa | ttctctgctaaaaacggg |
| -8409/+38EBS1mt | tccacgaaaaaggcttcg | cgaagcctttttcgtgga |
| -8409/+38EBS2mt | ttacccgaaaaggagcat | atgctccttttcgggtaa |
| -8409/+38EBS3mt | agggcccaaaacagtcct | aggactgttttgggccct |
| -8409/+38EBS4mt | agtccggcgaaaagggcc | ggcccttttcgccggact |
| -8409/+38EBS5mt | acacacacaaaaagtccg | cggactttttgtgtgtgt |
| -8409/+38EBS6mt | cgctgacggatcaccgtt | aacggtgatccgtcagcg |
| -8409/+38EBS7mt | cgctgacagatccccgtt | aacggggatctgtcagcg |

Linker scanning – region A.

| forward primer | tacgtacgtacgtacgtacgcaacaaccccctcctccaca |
| --- | --- |
| reverse primer | cgtacgtacgtacgtacgtactagccctgaagcagtcaga |
| ScanA_1_fwd | tacgtacgtacgtacgtacggagccaggaatttccggcca |
| ScanA_1_rev | cgtacgtacgtacgtacgtatgtggtccaggacctgtggg |
| ScanA_2_fwd | tacgtacgtacgtacgtacgttgtccagactagagctggc |
| ScanA_2_rev | cgtacgtacgtacgtacgtaaggcttcagggtcaggcacg |
| ScanA_3_fwd | tacgtacgtacgtacgtacgtcaggaagccgtgatcttca |
| ScanA_3_rev | cgtacgtacgtacgtacgtatggccggaaattcctggctc |
| ScanA_4_fwd | tacgtacgtacgtacgtacggaaaagggtgatattggggg |
| ScanA_4_rev | cgtacgtacgtacgtacgtagccagctctagtctggacaa |
| ScanA_5_fwd | tacgtacgtacgtacgtacgaggggctttggggacatcct |
| ScanA_5_rev | cgtacgtacgtacgtacgtatgaagatcacggcttcctga |
| ScanA_6_fwd | tacgtacgtacgtacgtacgggctgagtccagaaggggcc |
| ScanA_6_rev | cgtacgtacgtacgtacgtacccccaatatcacccttttc |

Linker scanning – region D.

| ScanD_2_fwd | tacgtacgtacgtacgtacgcaacaaccccctcctccaca |
| --- | --- |
| ScanD_2_rev | cgtacgtacgtacgtacgtactagccctgaagcagtcaga |
| ScanD_3_fwd | tacgtacgtacgtacgtacggtttggtcagcccccctccc |
| ScanD_3_rev | cgtacgtacgtacgtacgtaattccctgggactccagggt |
| ScanD_4_fwd | tacgtacgtacgtacgtacgttaatgacctcctcagcccc |
| ScanD_4_rev | cgtacgtacgtacgtacgtatgtggaggagggggttgttg |
| ScanD_5_fwd | tacgtacgtacgtacgtacgttggcctcctgtttgttcgc |
| ScanD_5_rev | cgtacgtacgtacgtacgtagggaggggggctgaccaaac |
| ScanD_6_fwd | tacgtacgtacgtacgtacgtgacggatccccgttatcag |
| ScanD_6_rev | cgtacgtacgtacgtacgtaggggctgaggaggtcattaa |
| ScanD_7_fwd | tacgtacgtacgtacgtacgcagagaacacacacaggaag |
| ScanD_7_rev | cgtacgtacgtacgtacgtagcgaacaaacaggaggccaa |
| ScanD_8_fwd | tacgtacgtacgtacgtacgtccggcgggaagggcccatc |
| ScanD_8_rev | cgtacgtacgtacgtacgtactgataacggggatccgtca |
| ScanD_9_fwd | tacgtacgtacgtacgtacgccagtcctgatttacccggg |
| ScanD_9_rev | cgtacgtacgtacgtacgtacttcctgtgtgtgttctctg |
| ScanD_10_fwd | tacgtacgtacgtacgtacgatggagcataaaaagccacc |
| ScanD_10_rev | cgtacgtacgtacgtacgtagatgggcccttcccgccgga |
| ScanD_11_fwd | tacgtacgtacgtacgtacgcttcttcacccgggaggagc |
| ScanD_11_rev | cgtacgtacgtacgtacgtacccgggtaaatcaggactgg |
| ScanD_12_fwd | tacgtacgtacgtacgtacgctctgtgcactggcaggctg |
| ScanD_12_rev | cgtacgtacgtacgtacgtaggtggctttttatgctccat |
| ScanD_13_fwd | tacgtacgtacgtacgtacgctgtgcagtccacgaggaag |
| ScanD_13_rev | cgtacgtacgtacgtacgtagctcctcccgggtgaagaag |
| ScanD_14_fwd | tacgtacgtacgtacgtacggcttcggtcgagatctgcga |
| ScanD_14_rev | cgtacgtacgtacgtacgtacagcctgccagtgcacagag |
