# Supplementary material for: VE-statin/egfl7 Expression in Endothelial Cells Is Regulated by a Distal Enhancer and a Proximal Promoter under the Direct Control of Erg and GATA-2
Source: PLoS One. 2010 Aug 16;5(8):e12156. doi: 10.1371/journal.pone.0012156 (PMC2922337; doi:10.1371/journal.pone.0012156)
Supplement: Table S2 — Primers and conditions used in qPCR analysis following ChIP. Primers are listed in the 5′→3′ orientation. (0.03 MB DOC) [file pone.0012156.s009.doc]

| **forward primer** | **reverse primer** | **Position on the gene** | **Annealing temperature (°C)** |
| --- | --- | --- | --- |
| atgagctcacagcttggagggccggata | atatctcgagttttctgaagatcacggc | -7961/-7686 | 62 |
| gctctaaatggggcctgggatgttga | gggctgtgaaggggtgggggatgttt | -4655/-4434 | 58 |
| accccctttttgctgtttag | agagatggccttcctgtttgtc | -3080/-2872 | 58 |
| catgttggagggagggtgccgaagag | atggaaagggatgggagggataaagt | -1153/-867 | 58 |
| ggtctcctcattagtagtga | tatctcgtctttggtgacac | +550/+868 | 60 |
| gtcacagcaccccatagcgtagc | cgactcaacagccccctacc | +3108/+3405 | 62 |
| agataccagaaagccctcactcc | gctcagctggtcccttatcaac | +4823/+5003 | 62 |
| cataacatagctggttcttgttc | gataagtgtagctgcctggtg | *ß-globin* | 55°C |
| gaatgctctggcagaagcttagagc | aggcaggttttccaactggccc | *VE-cadherin* | 62°C |
