# Supplementary material for: VE-statin/egfl7 Expression in Endothelial Cells Is Regulated by a Distal Enhancer and a Proximal Promoter under the Direct Control of Erg and GATA-2
Source: PLoS One. 2010 Aug 16;5(8):e12156. doi: 10.1371/journal.pone.0012156 (PMC2922337; doi:10.1371/journal.pone.0012156)
Supplement: Table S3 — Primers used in qPCR. Primers are listed in the 5′→3′ orientation. (0.05 MB DOC) [file pone.0012156.s010.doc]

| **Gene name(s)** | **forward primer** | **reverse primer** |
| --- | --- | --- |
| *ets1* | ggagagccagtcgtggtaaact | ggtgaggcggtcacaactatc |
| *tel/etv6* | cccgccgagcgtttct | aattcgttcctgcttaatgctagac |
| *ﬂi-1* | tcaaccagccagtgagagtcaat | caccggagactccctggat |
| *erg* | tgccgacattcttctctcacat | tcaacgtcatcggaagtcagat |
| *net/elk3/sap2* | agtcctgccaggctgcaa | cggcatgtgaccgttgag |
| *elf1* | agcagcagcttggtgatcct | tgtcattaggctcttccacacaa |
| *etv1/er81* | ccggcgatgaactatgacaa | tctccagccacct4gcat |
| *elf2/nerf2* | accagacagccacgaacca | tggtgactgctgggtctttg |
| *elf3/ese1* | ggcactgaagacttggtgttga | agcttgccttctctggacctt |
| *gabpα* | taccaaggaatggagccaaagt | ggtgggcatctggatcgat |
| *etv5/erm* | cagccaatgcccacttcat | agcaacctcttccggttctatg |
| *pea3/etv4* | tggagcggaggatgaaagg | cgggagatttgctgcagaag |
| *sam/pdef* | tcaagccccacagctatgg | tttgaagatgcctttctccttgt |
| *ets2* | tctcagtgaagatcagacactccaa | ccgagtcatgggagacagaatc |
| *pe1/etv3* | acaacaagaggatccttcataaaaca | ccacaccactcgagcgaat |
| *fev* | ctcccagcccctgctgat | agaaactgccacaactggatctg |
| *etv2/estrp71* | gactgggagcggaatttggt | tgtgatcggtgtgtcctcttg |
| *elk4/sap1* | gccgagccctgcgatac | acacaaacttctgaccattcact |
| *elk1* | cctgctccccacacatacct | ctggacggaaactggaagga |
| *ehf* | cagttgcagagtcacctgatatga | tgcctcttgggttgtgcttt |
| *elf4* | agaccaagaacaaccgaagtacct | gtgctgcctttgccatcttt |
| *notch1* | ggccgccttcgtgctcctgtt | gctggcgccctggtagatgaagtc |
| *agpat2* | gcggcgctgctgctgctgttg | tctggcttgctggcggttgatgaa |
| *gata-1* | gtcagaaccggcctctcatc | gtgcctgcccgtttgc |
| *gata-2* | cacctgttgtgcaaattgtcaga | ggatcccttccttcttcatggt |
| *gata-3* | gaaccggccccttatcaag | acagttcgcgcaggatgtc |
| *gata-4* | gggccaaccctggaagac | gacacactctctgccttctgagaa |
| *gata-5* | caaagaaccctgccaaaatca | gactggactctgccttcaaagtg |
| *gata-6* | agcgcgtgccttcatca | Gtagtggttgtggtgtgacagttg |
